# Hyperspectral imaging: a novel approach for plant root phenotyping

**DOI:** 10.1186/s13007-018-0352-1

**Published:** 2018-10-03

**Authors:** Gernot Bodner, Alireza Nakhforoosh, Thomas Arnold, Daniel Leitner

**Affiliations:** 1 Division of Agronomy, Department of Crop Sciences, University of Natural Resources and Life Sciences, Vienna (BOKU), Konrad Lorenz-Straße 24, 3430 Tulln an der Donau, Austria; 2 Carinthian Tech Research AG, Europastraße 12, High Tech Campus Villach, 9524 Villach/St. Magdalen, Austria; 3 Agriculture and Agri-Food Canada, Brandon Research and Development Centre, Brandon, MB R7A 5Y3 Canada; 4 Computational Science Center, University of Vienna, Oskar-Morgenstern-Platz 1, 1090 Vienna, Austria; 5 Simulationswerkstatt, Ortmayrstrasse 20, 4060 Leonding, Austria

**Keywords:** Hyperspectral imaging, Image processing, Phenotyping, Root decomposition, *Triticum durum*

## Abstract

**Background:**

Root phenotyping aims to characterize root system architecture because of its functional role in resource acquisition. RGB imaging and analysis procedures measure root system traits via colour contrasts between roots and growth media or artificial backgrounds. In the case of plants grown in soil-filled rhizoboxes, where the colour contrast can be poor, it is hypothesized that root imaging based on spectral signatures improves segmentation and provides additional knowledge on physico-chemical root properties.

**Results:**

Root systems of *Triticum durum* grown in soil-filled rhizoboxes were scanned in a spectral range of 1000–1700 nm with 222 narrow bands and a spatial resolution of 0.1 mm. A data processing pipeline was developed for automatic root segmentation and analysis of spectral root signatures. Spectral- and RGB-based root segmentation did not significantly differ in accuracy even for a bright soil background. Best spectral segmentation was obtained from log-linearized and asymptotic least squares corrected images via fuzzy clustering and multilevel thresholding. Root axes revealed major spectral distinction between center and border regions. Root decay was captured by an exponential function of the difference spectra between water and structural carbon absorption regions.

**Conclusions:**

Fundamentals for root phenotyping using hyperspectral imaging have been established by means of an image processing pipeline for automated segmentation of soil-grown plant roots at a high spatial resolution and for the exploration of spectral signatures encoding physico-chemical root zone properties.

**Electronic supplementary material:**

The online version of this article (10.1186/s13007-018-0352-1) contains supplementary material, which is available to authorized users.

## Background

The root system is fundamental for plant physiological and ecosystem functioning [39, 50, 61]. Better understanding of root and rhizosphere processes can therefore essentially contribute to enhance resource efficiency in crop production and sustainable soil management [26, 38, 63, 68].

Advances in root system and rhizosphere management critically depend on appropriate measurement methods, making the plants’ “hidden half” [5] accessible to visualization and quantification. Traditionally root research relied on destructive and highly laborious methods (e.g. coring, profiling; [67]). Since the 1990 tie non-destructive imaging methods have become increasingly popular in plant sciences [18, 36]. Imaging of plant root systems has evolved into two directions: (1) deep phenotyping using high resolution 3D methods for small scale processes (e.g. X-ray computer tomography, [45]; magnetic resonance imaging, [69]), and (2) high-throughput phenotyping using optical methods [17]. In spite of their different focus, there is a trend towards approximation owing to technological advance. E.g. current μCT devices yet allow imaging of larger soil volumes up to a range of 1000 cm^3^ at high resolutions in reasonable measurement time for phenotyping purposes (range: hour; [40]). On the other hand high-throughput applications, traditionally restricted to RGB imaging, are moving towards wider spectral ranges where chemical imaging of rhizosphere components is possible [12, 54]. Nakaji et al. [49] were the first to demonstrate application of hyperspectral imaging (480–972 nm) in order to discriminate between living, senescent and dead roots, leaf debris and soil. For leave tissues, Pandey et al. [51] showed that using an extended hyperspectral range from 550 to 1700 nm enables an accurate prediction of leaf water content and nutritional status.

Most root phenotyping approaches rely on seedling/juvenile plants grown in artificial media such as agar [27], germination paper [33] or hydroponics [10] to facilitate imaging. Extrapolation from such systems towards natural growing conditions has been questioned [75, 80]. Therefore larger soil-filled rhizobox systems have been established for field-near root imaging [37, 47]. RGB root imaging of rhizobox grown plants has been successfully used to characterize distinctive root architectures in breeding germplasm as well as the modification of root architecture in response to variable soil conditions (temperature, water and nutrient supply, compaction; e.g. Price et al. [57], Nagel et al. [46, 48]).

RGB root phenotyping however requires sufficient root-soil colour contrast for (automated) segmentation which is challenging in case of bright coloured soils and/or dark coloured roots. Pierret [54] was the first to suggest spectral imaging of rhizoboxes as a promising advance for root research. Hyperspectral data might overcome problems of root distinction from soil background in the RGB colour space. Furthermore spectral reflectance encodes material properties that can be captured via advanced statistical models (chemometrics; [76]), potentially providing a tool to simultaneously measure root architecture with relevant physico-chemical root zone properties (e.g. water content). Close-range spectral imaging has already been successfully applied in phenotyping aboveground plant parts [43]. However, besides the system described by Nakaji et al. [49], there are still no established spectral imaging systems and data processing pipelines for root phenotyping.

Here a first comprehensive methodological description and evaluation of hyperspectral root phenotyping is presented. Acquisition of high resolution hyperspectral root images and subsequent image processing is described in detail. Root segmentation based on spectral information is compared with standard colour based segmentation. Application of the novel method is exemplified for identification of spectrally distinctive regions within the root and spectral changes during root decay. Classification of such chemometrically distinctive root regions is an important information when aiming to understand possible functional differences between root axes in space and time. The aim here is to establish the fundamentals of hyperspectral imaging for plant root phenotyping and reveal its potential as a novel tool for improved root system and rhizosphere characterization.

## Methods

### Rhizobox setup

Plants were grown in rhizoboxes (30 × 100 × 1 cm) consisting of a grey PVC back-plate and side frames (thickness 3 cm), and a mineral glass front (thickness 8 mm) fixed by metal angle bars to the sides. At the bottom, drainage holes were drilled into the frame.

Boxes were filled with two substrates to compare different background effects on imaging, i.e. dark topsoil and bright subsoil from a calcareous chernozem [23] sieved to an aggregate size < 2 mm. Table 1 gives an overview of basic soil properties. Soil moisture was adjusted to a matrix potential of h = − 100 cm (drained upper limit for a rhizobox of height/gravitational potential of 100 cm) for a well-watered treatment and h = − 1000 cm for a water limited treatment (retention curve of the two substrates, Additional file 1).

**Table 1:** Basic soil properties (texture, C_org_), water content (WC) at a matrix potential of h = − 100 cm (well-watered) and h = − 1000 cm (water-limited), and soil colour (Munsell colour code)

Type	Soil properties		Colour (hue, value, chroma)	
			Dry	Wet
Topsoil	Sand (g g^−1^)	21.9	10 YR 3/2	10 YR 2/2
	Silt (g g^−1^)	61.2		
	Clay (g g^−1^)	16.9		
	C_org_ (g g^−1^)	2.0		
	WC_100 cm_ (cm^3^ cm^−3^)	0.27		
	WC_1000 cm_ (cm^3^ cm^−3^)	0.18		
Subsoil	Sand (g g^−1^)	26.4	2.5 Y 5/4	2.5 Y 4/6
	Silt (g g^−1^)	69.6		
	Clay (g g^−1^)	4.0		
	C_org_ (g g^−1^)	0.8		
	WC_100 cm_ (cm^3^ cm^−3^)	0.33		
	WC_1000 cm_ (cm^3^ cm^−3^)	0.13		

10 g of slow release NPK fertilizer (16% N, 2.6% P, 11.6% K) were mixed into the soil. Rhizoboxes were planted directly with one presoaked seed from *Triticum turgidum* L. subsp. *durum* cv. Floradur at a depth of 2 cm below soil surface. The glass surface/root zone was darkened with a black plate and the slit at the top of the boxes was closed with PVC foam to minimize evaporation. Boxes were positioned at 45° angle into a metal frame and transferred into a growth chamber (Light intensity 250 mmol m^2^ s^−1^ by six Atum Photon 270 LED; day/night 14 h/10 h; temperature 23 °C/16 °C; Relative humidity 60%).

The experimental design was a CRD with four replicates and substrate (bright vs. dark) and moisture (dry vs. moist) as main factors (i.e. 16 rhizoboxes). Plants were watered every third day upon weighing to keep the initial moisture level. Imaging was done for fully developed root systems (BBCH 44), i.e. when reaching the bottom part of the boxes and showing all types of roots (primary/basal roots, shoot borne roots, laterals; [81]). At this stage, 2.2% of total root length was < 0.2 mm and 48.7% < 0.5 mm diameter.

For the analysis of root decay we followed Nakaji et al. [49], cutting the shoot and subsequently imaging the upper third of the rhizobox (with primary, shoot-borne and lateral roots) at 14, 28, 47, 94, 101 and 201 days after cutting.

### Image acquisition setup

**Fig. 1:**
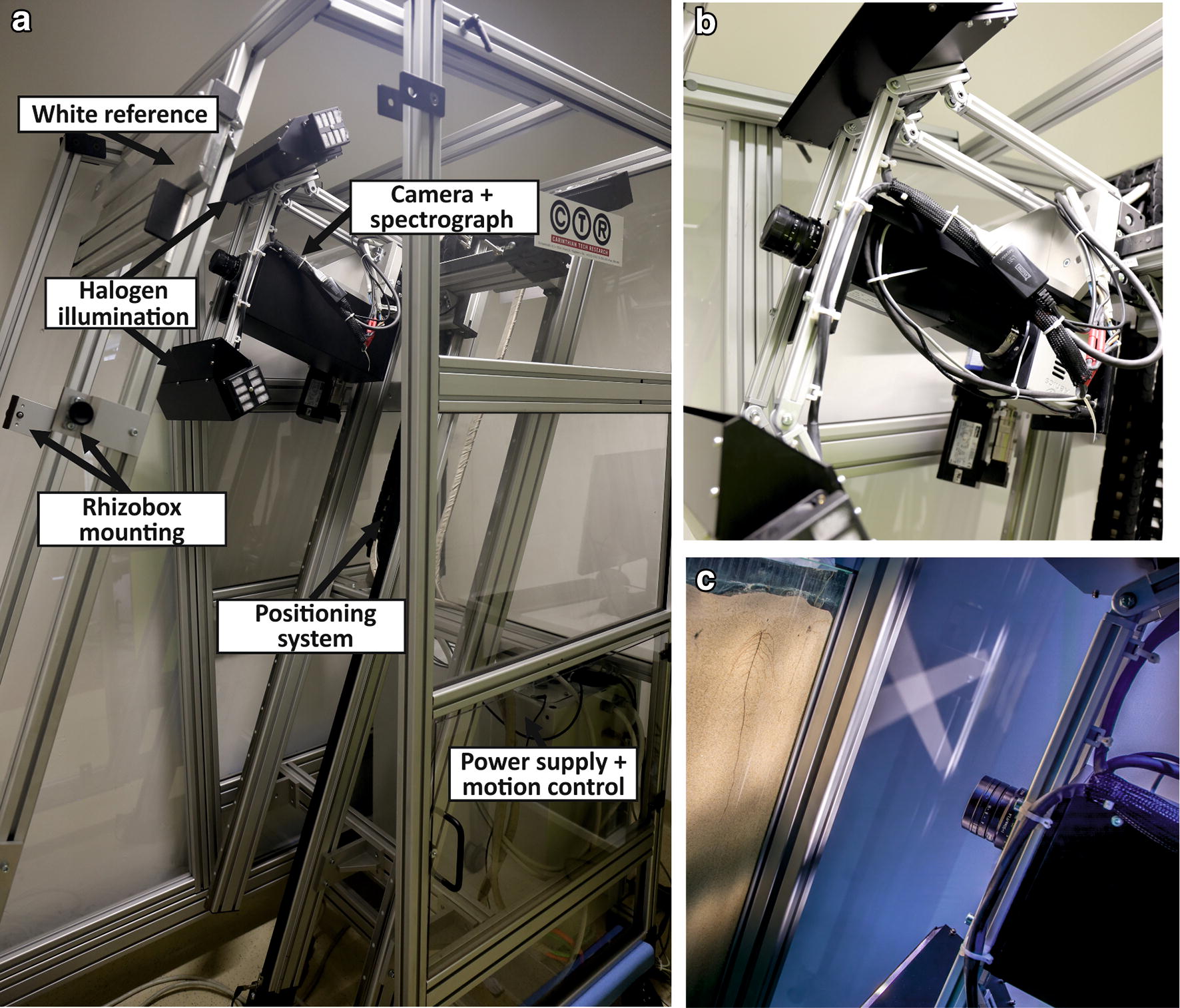
Measurement setup of the hyperspectral root imaging system, with **a** system components, **b** close-up of the camera and illumination, and **c** scanning of a rhizobox

The hyperspectral root imaging system (Fig. 1) consists of four main components: (1) A halogen line illumination (45°/− 45°) that provides a homogeneous light sources for imaging the rhizobox; (2) an image spectrograph (ImSpector N17E, Specim, FI) that splits the incoming light into 256 spectral bands between 900 and 1700 nm; (3) a thermo-electrically cooled 14-bit monochrome NIR camera (Xeva, Xenics, BE) that records the spectral NIR bands (900–1700 nm) for each pixel. The resolutions of the camera sensor is 320 by 256 pixels and the frame rate is 100 Hz. The spectral resolution of the imaging system is 3.1 nm (256 bands between 900 and 1700 nm). Due to low sensitivity of the detector chip at the lower edge, resulting in noisy spectra, the spectral bands were limited to the wavelength range of 1000–1700 nm.

The distance between the front lens and the rhizobox is 18 cm with a perpendicular position of the rhizobox with respect to the camera. A white standard (Spectralon tile) is positioned at the top of the rhizobox at the same angle and distance and imaged before each scan. The white standard is covered by the same type of glass as used for the rhizoboxes to compensate for the light transmission path through the glass upon normalization with the white and dark standards (see “Image preparation” section).

A hyperspectral rhizobox image (with x, y as spatial dimensions and z as spectral dimension) is obtained using a push-broom approach, i.e. the imaging system (illumination + spectrograph + camera) is moving in the y-direction while continuously capturing line scans in the x-direction of the rhizobox which are projected into the spectrograph and recorded by the camera. With a field of view (FOV) of 3 cm, maximum spatial resolution is 0.1 mm. A 50 mm lens is used to achieve this image resolution at 10 mm s^−1^ scanning speed. Via a two-axis positioning system, moving the imaging system up and down, spectral line scans (strides) of 3 cm width with 10% overlap between adjacent strides are acquired. Each stride is saved separately in a compressed file format (SIF) to reduce the size of the files. The single strides are composed to a full rhizobox image during image processing (see “Image processing” section).

Prior to scanning, camera integration time has to be adjusted to the background colour for optimizing image quality. The optimum camera integration time is obtained using the Xenics Xeneth camera software and targeting a bright object (i.e. root) on the rhizobox; it is adjusted in a way that approximately 85% of the full dynamic range of the imaging system are used. Exceeding this maximum will result in data losses during image acquisition, while on the other hand low integration time does not make use of the full capacity of the camera. Here an integration time of 4500 µs for the darker topsoil filled rhizoboxes and 3500 µs for the brighter subsoil filled rhizoboxes were used respectively.

The setup was developed as a prototype for root phenotyping by Carinithian Tech Research (CTR). It is located in a closed room to avoid influence of stray light during image acquisition and keeping the measurement device at stable temperature (20 °C) and air humidity (40%).

### Image processing

**Fig. 2:**
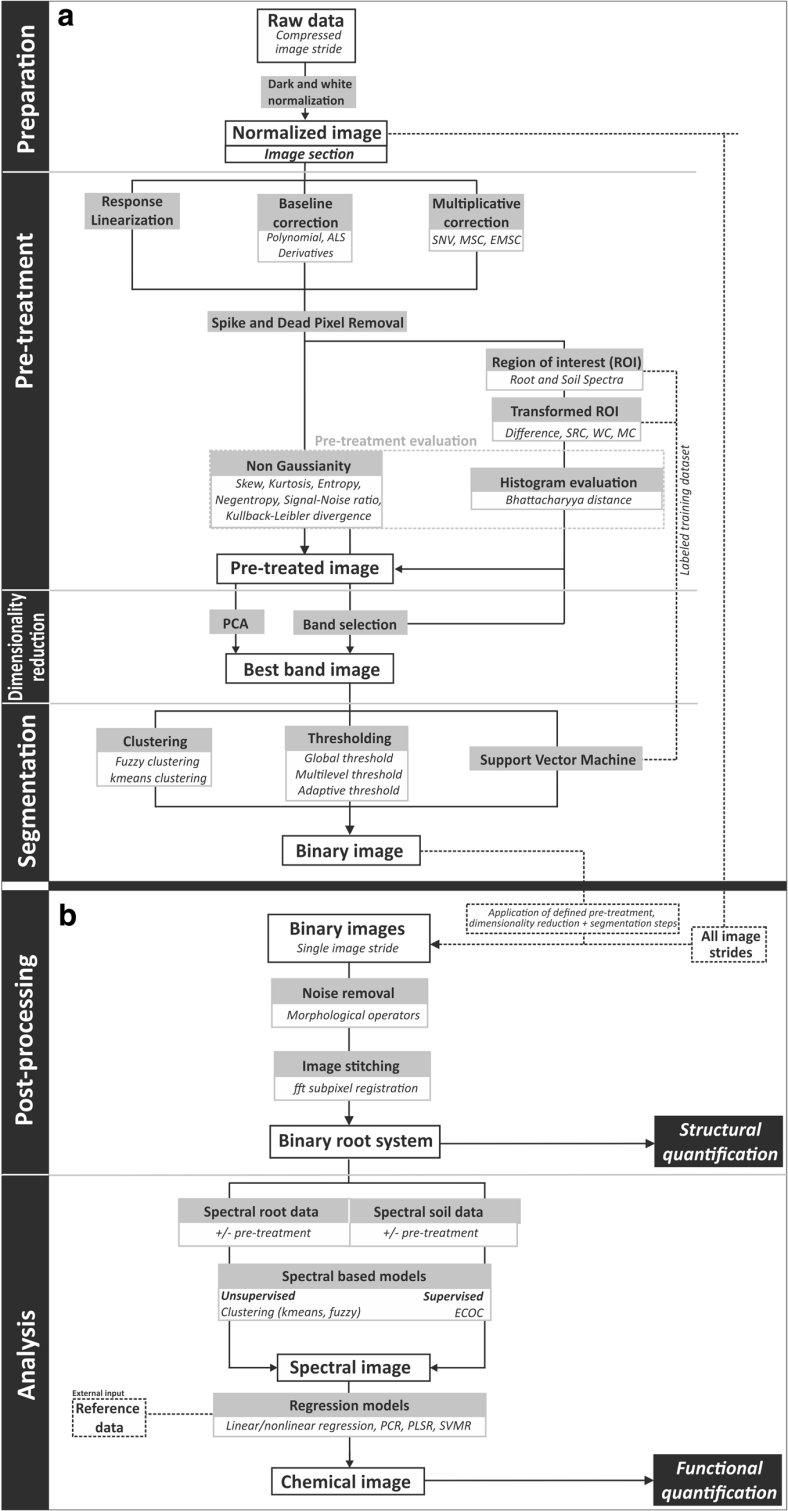
Scheme of the image processing pipeline developed for root segmentation and chemometric analysis of root and soil properties from hyperspectral images. Implemented approaches for **a** pre-processing and **b** post-processing and image analysis. Abbreviations: *ALS* asymmetric least squares correction, *SNV* standard normal variate, *MSC* multiplicative scatter correction, *EMSC* extended multiplicative signal correction, *SCR* simple contrast ratio, *WC* Weber contrast, *MC* Michelson contrast, *PCA* principal component analysis, *fft* fast Fourier transform, *ECOC* error correcting output codes, *PCR* principal component regression, *PLSR* partial least squares regression, *SVMR* support vector machine regression. (The Matlab scripts can be obtained from the corresponding author upon request.)

Figure 2 schematically presents the image processing pipeline. All steps are performed via Matlab scripts (Matlab version R2018a) on an Intel Core i7-6700 PC with 40 GB RAM. Scripts are available from the authors upon request.

#### Image preparation

Absolute reflectance of the raw image (R_i,λ_) is normalized at each wavelength band λ for each pixel *i* following Eq. (1) and using the dark (D_i,λ_) and white (W_i,λ_) standards acquired before each scan. The dark standard corrects for the wavelength dependent dark current of the NIR camera, while the white standard represents maximum (100%) reflectance. The normalized image (N_i,λ_) is thus obtained by:

1 $$N_{i,\uplambda } = \frac{{R_{i,\uplambda } - D_{i,\uplambda } }}{{W_{i,\uplambda } - D_{i,\uplambda } }}$$

#### Pre-treatment

Following Esquerre et al. [16] three types of data pre-treatments are implemented, i.e. response linearization enhancing low reflectance regions, baseline correction to correct for offset and change in the baseline (polynomial detrending, derivatives, asymmetric least squares, ALS) and multiplicative correction (standard normal variate, SNV, multiplicative scatter correction, MSC, extended multiplicative signal correction, EMSC). For most pre-treatments the mda toolbox for Matlab [31] is used. EMSC is performed via the toolbox from Afseth and Kohler [1]. However, due to excessive computational time of MSC and EMSC for the large-size image data evaluated here, they were not included in further evaluations.

Dead pixels and spikes, containing no information and potentially disturbing segmentation and analysis, are identified via the standard deviation matrix of difference spectra N_i,λ_ − N_i,λ+1_. A threshold is visually defined from the standard deviation histogram and the respective pixels are black masked (i.e. assigned a value of zero over all wavelengths; Vidal and Amigo [72] and Dorrepaal et al. [13]).

#### Pre-treatment method selection and dimensionality reduction

Identification of adequate pre-treatment methods and spectral bands maximizing feature distinction require selection criteria. A first selection approach targets the entire image: it combines a set of Non-Gaussian indicators (skewness, kurtosis, entropy, negentropy, signal-to-noise ratio, Kullback–Leibler divergence) that measure deviation from random white noise [8, 21]. As single indicators can lead to different results [19] an average rank of all information criteria is used to select the top-ranking pre-treatment method.

The second approach is based on histogram evaluation [29]. Root (foreground) and soil (background) regions of interest (ROIs) are selected and the separation of their histogram peaks at each wavelength is quantified via Bhattacharyya distance [4]. Spectral transformations (differences spectra, N_i_,_λn_ − N_i_,_λm_, λ_m_ and λ_n_= 1…222) were evaluated with the ROI based histogram method only: due to sharply increasing dimensionality (222 × 222) transformation of entire images for the non-Gaussian indicator approach is not feasible for computational reasons.

The three best pre-treatment methods selected by each approach, i.e. (1) non-Gaussianity based, (2) Bhattacharyya distance based for (a) single spectra as well as (b) difference spectra, were further used for comprehensively testing the influence of pre-treatment on subsequent segmentation.

Simultaneously with pre-treatment evaluation, the most informative wavelengths within the pre-treated images are extracted, while removing all noisy bands. Here the ten best bands with highest frequency of occurrence among the applied evaluation approaches were selected. For comparison PCA was used as reference dimensionality reduction method. Depending on the segmentation algorithm either 2D or 3D data can be used. 3D images contained all ten selected bands, 2D data contained one spectral dimension only: reduction to a single spectral dimension was done via (1) averaging over the ten best bands, (2) extracting the single most informative band and (3) the principal component with highest feature contrast.

#### Image segmentation

Segmentation is performed with unsupervised (multilevel thresholding, k-means clustering, fuzzy clustering) and supervised (two-class support vector machine) techniques. The implemented fuzzy clustering algorithm was presented by Li et al. [34], while all other algorithms are adapted from standard Matlab scripts. Evaluation of segmentation quality was done based on obtained root length in relation to a manually tracked reference length and image skewness: misclassification can increase false negatives (root pixels wrongly classified as soil background) resulting in a conservative segmentation with low noise (high skewness) at the cost of underestimation of root length; false positives (soil pixels wrongly classified as root) on the contrary result in a noisy image (low skewness) and corresponding overestimation of root length.

#### Post-processing

Post-processing comprises stitching of all strides to an entire binary root system image and removal of remaining noise in the image. Automatic stitching is done with an image registration algorithm based on cross-correlation of image pixels in Fourier domain [22]. Noise removal is performed by filtering for small circular objects using region property analysis with a manually set minimum extent.

### Chemometric root analysis

Chemometric models delimit physico-chemically distinctive regions within the segmented root and/or soil domains based on their specific spectral patterns. Also here unsupervised (fuzzy and k-means clustering, PCA) and supervised (Multiclass error-correcting output codes, ECOC, with optional classifiers, e.g. support vector machine, k-nearest neighbour, discriminant analysis, decision tree) approaches are implemented.

With directly measured reference data spectral regression models can be applied. Scripts for linear and non-linear univariate regression, partial least squares regression (PLSR), principal component regression (PCR) and support vector machine regression (SVMR) were included.

The application of chemometric models is exemplified for the analysis of spectrally distinctive radial (centre to border; supervised ECOC model) pattern on the roots, as well as for root decay following clipping of the shoot (non-linear regression model). For radial differentiation the image was shape-corrected via ALS baseline correction to exclude confounding effects from surface geometry of spherical objects [58] and dimensions were reduced to the first five principal components. Eight radial classes from root centre (0–0.1 mm) to border pixels at 2 pixel increments were defined and the respective pixels were labelled accordingly. An ECOC model with a decision tree classifier [6] was trained with 50% of pixels from the eight radial classes and validated with the remaining 50% of pixels.

For root decay, the most distinctive wavelength separating spectra (untransformed, first derivative, single wavelength and wavelength differences) at different times after clipping was identified via Bhattacharyya distance. Spectral reflectance was then related to decay time via an exponential model and subsequently mapped on each root pixel.

### Imaging data

Determination of an adequate image processing strategy (pre-treatment, dimensionality reduction, segmentation) was done on topsoil and subsoil image sections (320 × 3000 pixel, 222 wavebands from the densely rooted upper part of rhizoboxes, i.e. one central stride with one third of the vertical pixels). The reduced file size of 9.4 GB ensured comparatively short calculation time for the single steps, required for evaluating all processing options. The resulting image processing strategy is then applied to all rhizoboxes of the same treatment resulting in binary root system images for structural quantification. With the 40 GB RAM PC used here the Matlab based processing pipeline takes about 40 min to obtain a binary rhizobox image from the spectral raw data.

In this study structural quantification was done with WinRhizo (Version 2013; Regent Inc.) for root length only. Visible root length from spectral segmentation was compared to colour based segmentation from RGB images. RGB images were taken with a Canon EOS 6D camera (resolution 5472 × 3648 pixel) after the spectral scans. Images were converted to TIFF-files and segmented using WinRhizoPro based on five distinctive colour classes for root and soil respectively. Manually tracked roots on RGB images were used as reference. Manual tracking was done in CorelDraw (Version X7) using a graphic tablet (Wacom Intuos^®^pro) and zooming into the image (500% magnification) to capture all root axes visible on the glass surface.

Visible length was also compared to total length, including the non-visible roots, obtained at the end of the experiment after separating (washing) roots from soil following Himmelbauer et al. [25].

Chemometric analysis of distinctive root regions and root decay is exemplified with a segmented root image from the well-watered topsoil treatment.

### Statistical evaluation

The influence of pre-treatment, dimensionality reduction and segmentation methods was evaluated by analysis of variance. The dependent variable was root length, while skewness of the segmented binary image was used as covariate capturing the influence of misclassified pixels. Cases where segmentation failed (noise threshold: skewness < 2.5; no visual identification of the root system) were excluded. Evaluation of spectral versus colour based segmentation in relation to the manually tracked reference was done by regression analysis with zero intercept. Slopes were compared statistically following Sawand [59] to reveal whether the two segmentation approaches differed significantly in capturing visible root length. All statistical analyses were performed in SAS Version 9.4 using PROC MIXED for covariance analysis, PROC REG for regression analysis and PROC GLM for slope comparison.

## Results

### Image processing strategy

Hyperspectral rhizobox images with 222 bands in the spectral range of 1000–1700 nm and a spatial resolution of 0.1 mm result in an image size of 42.3 GB. Using a representative image section of 320 × 3000 pixels near the plant base, file size was decreased to 9.4 GB for deriving an adequate image processing strategy, while reducing computational time sufficiently to test several possible combinations of processing steps (pre-treatment, dimensionality reduction, segmentation) on a standard PC.

#### Spectral pre-treatment

Image pre-treatment corrects uneven surface morphology due to soil aggregates, resulting in inhomogeneous illumination with increased reflectance scattering [16]. In total 16 pre-treatment approaches were evaluated via non-Gaussian indicators (whole image section) and Bhattacharyya distance (root and soil ROIs; 0.6% of image pixels). Bhattacharyya distance was also calculated for difference spectra (graphical examples, Additional file 2). Evaluation results are given in Table 2.

**Table 2:** Selection of best pre-treatment methods (combination of linearization, de-trending and multiplicative correction) from non-Gaussian information indicators and Bhattacharyya distance

	Linearization	De-trending	Multiplicative correction	Non-Gaussian measure rank	Bhattacharyya distance R (rank)	Bhattacharyya distance R_i_ − R_j_ (rank)
*Topsoil*						
1	Non*	Non	Non	15	0.28 (15)	*9.40 (3)*
2	Non	Polynomial	Non	16	0.12 (16)	*9.94 (1)*
3	Non	Derivative	Non	6	*4.89 (2)*	8.24 (4)
4	Non	ALS	Non	4	4.05 (5)	*9.86 (2)*
5	Non	Non	SNV	14	4.55 (4)	5.21 (12)
6	Non	Polynomial	SNV	11	*9.05 (1)*	5.52 (9)
7	Non	Derivative	SNV	10	1.78 (12)	3.81 (15)
8	Non	ALS	SNV	9	2.16 (11)	4.07 (14)
9	Log(1/R)	Non	Non	8	0.30 (14)	5.30 (11)
10	Log(1/R)	Polynomial	Non	7	1.00 (13)	5.46 (10)
11	Log(1/R)	Derivative	Non	*1*	2.57 (10)	4.94 (13)
12	Log(1/R)	ALS	Non	*2*	*4.63 (3)*	3.67 (16)
13	Log(1/R)	Non	SNV	12	3.08 (7)	6.77 (7)
14	Log(1/R)	Polynomial	SNV	13	3.65 (6)	6.46 (8)
15	Log(1/R)	Derivative	SNV	5	3.03 (8)	7.87 (5)
16	Log(1/R)	ALS	SNV	*3*	2.69 (9)	7.14 (6)
*Subsoil*						
1	Non	Non	Non	13	0.26 (15)	3.18 (16)
2	Non	Polynomial	Non	14	0.11 (16)	3.48 (15)
3	Non	Derivative	Non	15	2.76 (12)	7.04 (11)
4	Non	ALS	Non	10	2.56 (13)	6.20 (12)
5	Non	Non	SNV	9	3.68 (9)	9.36 (6)
6	Non	Polynomial	SNV	7	3.87 (8)	9.88 (5)
7	Non	Derivative	SNV	16	3.89 (7)	4.52 (14)
8	Non	ALS	SNV	12	5.84 (6)	7.39 (10)
9	Log(1/R)	Non	Non	8	*7.44 (2)*	*11.95 (3)*
10	Log(1/R)	Polynomial	Non	11	1.10 (14)	10.61 (4)
11	Log(1/R)	Derivative	Non	5	3.61 (10)	8.87 (7)
12	Log(1/R)	ALS	Non	*2*	5.91 (5)	*13.62 (1)*
13	Log(1/R)	Non	SNV	*3*	*6.60 (3)*	8.19 (8)
14	Log(1/R)	Polynomial	SNV	4	6.37 (4)	7.56 (9)
15	Log(1/R)	Derivative	SNV	6	3.36 (11)	5.07 (13)
16	Log(1/R)	ALS	SNV	*1*	*10.52 (1)*	*11.51 (2)*

For non-Gaussian measures the rank represents a mean of six different indicators; for ROIs (R, single wavelengths, R_i_ − R_j_, wavelength differences) the absolute value of Bhattacharyya distance and the ranking of methods (in brackets) is given. The three best pre-treatment methods suggested by each selection approach are marked in italic

* Non no treatment, Log(1/R) log-linearization, polynomial 2nd order polynomial centring, derivative 1st order Savitzky–Golay derivative, *ALS* asymptotic least square correction, *SNV* standard normal variate

The indicators did not point to a unique pre-treatment method to maximize distinction between root foreground and soil background. Particularly in the topsoil there was no significant correlation between ranks from non-Gaussian measures and Bhattacharyya distance. In the subsoil on the contrary correlation coefficients between the methods were significant with an *r* > 0.61.

Among the three best pre-treatment methods indicated by each of the information criteria, de-trending via ALS baseline correction occurred in 44% of all cases. For the subsoil log-linearization resulted in an average improved foreground–background distinction. Difference spectra increased the distinctiveness of image features (Bhattacharyya distance topsoil 3.0–6.5; subsoil 4.2–8.0). The three top-ranking pre-treatment approaches from each information criterion for topsoil and subsoil were further used in the evaluation of subsequent image processing steps.

#### Dimensionality reduction

**Fig. 3:**
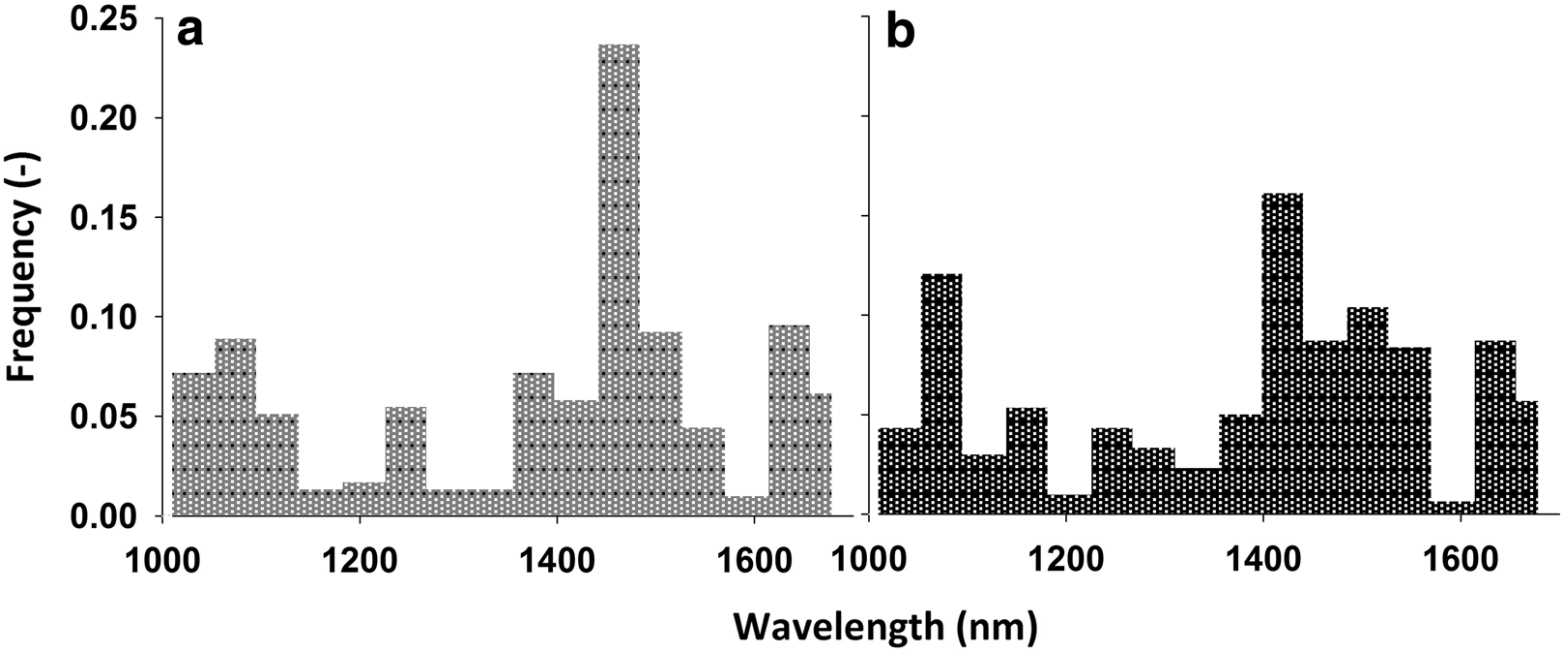
Frequency of occurrence of most informative bands in a topsoil (**a**) and subsoil (**b**) hyperspectral image according to different band selection approaches

Figure 3 gives an overview of the frequency of occurrence of wavelength regions with highest foreground–background contrast identified via band selection indicators (see movie Additional file 3). The highest spectral differentiation between root and soil occurred in the region from 1440 to 1480 nm (topsoil) and 1400 to 1440 nm (subsoil). A second distinctive region was found between 1050 and 1090 nm. Spearman rank correlation indicated that location of the most informative spectral bands in topsoil and subsoil was similar (r = 0.80, *P* < 0.001).

#### Segmentation algorithm

Four segmentation methods (fuzzy clustering, k-means clustering, multi-level thresholding and a two-class SVM) were evaluated with dimensionality reduced images. As expected there was a trade-off between segmented length and noise: the higher the number of pixels misclassified as roots the higher the noise from these sparsely distributed pixels (*cf.* Additional file 4). In the subsoil segmentation failed in 51.0% of the cases with high noise (skewness < 2.5) and no clearly identifiable root axes. This was mainly the case for thresholding (78.6%), while fuzzy clustering only failed in 17.9% of cases. In the topsoil segmentation only failed in 15.9% of all cases (thresholding 28.6%, fuzzy clustering 14.3%, k-means clustering 10.8%, SVM 10.0%).

Significant influences from previous processing steps on the final segmentation result are highlighted in Table 3. The highest significant interaction between processing steps was a soil type specific effect of pre-treatment and segmentation algorithm. Thus adequate pre-treatment methods strongly depended on the soil background as well as the subsequent segmentation method.

**Table 3:** Results of analysis of variance of various pre-processing steps on the segmented root length for two soil background materials (DF degrees of freedom)

Source	DF	F-value	*p* value
Soil	1	0.48	0.4978
Pre-treatment (Pre)	8	1.98	0.1088
Dimensionality reduction (Dim)	3	10.51	0.0003
Segmentation (Seg)	3	8.01	0.0013
Soil × Pre	9	6.42	0.0005
Soil × Dim	3	1.16	0.3532
Soil × Seg	3	2.51	0.0913
Pre × Dim	24	3.34	0.0055
Pre × Seg	24	5.83	0.0002
Dim × × Seg	4	1.47	0.2514
Soil × Pre × Dim	18	1.30	0.2940
Soil × Pre × Seg	*19*	*4.24*	*0.0017*
Pre × Dim × Seg	27	1.30	0.2826
Skewness	1	12.15	0.0026

The highest significant interaction is highlighted in italic

**Fig. 4:**
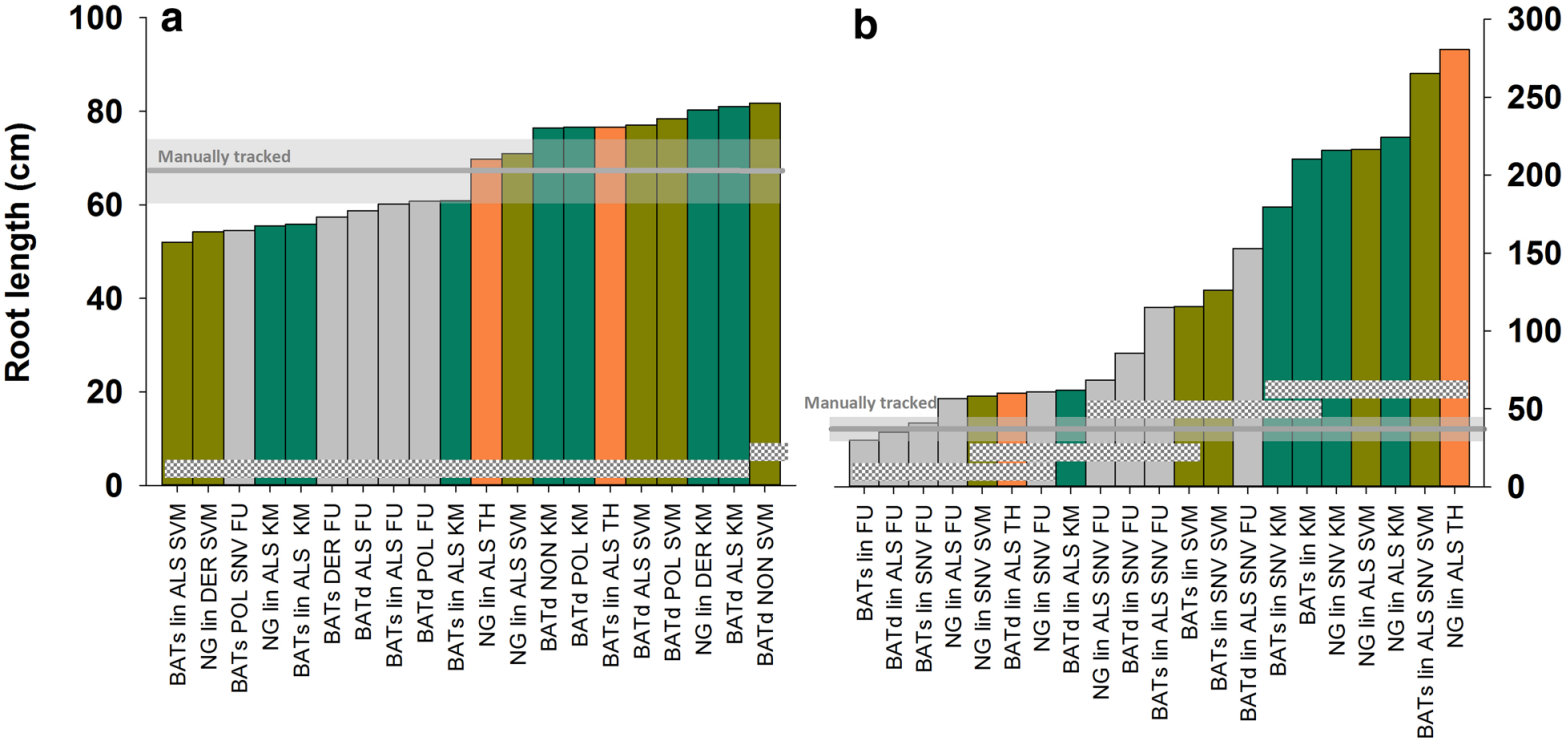
Root length for the twenty best combinations of pre-treatment, band selection and segmentation algorithm in the topsoil (**a**) and subsoil (**b**). The manually tracked reference length is shown with ± 10% margins (transparent grey area); grey dotted areas show the range of results without significant difference (Tukey, *p* < 0.005). NG refers to band selection via non Gaussian measures, BAT to Bhattacharyya distance based selection for single wavelength (BATs) and wavelength differences (BATd); for pre-treatments cf. Table 1; FU (grey) fuzzy clustering, TH (orange) thresholding, SVM (brown) support vector machine classification, KM k-means clustering (turquoise)

Figure 4 shows the resulting mean comparison (Tukey test) for the highest significant interaction (Soil × Pre × Seg). For clarity only the twenty segmentation results next to the manually tracked reference are plotted.

**Fig. 5:**
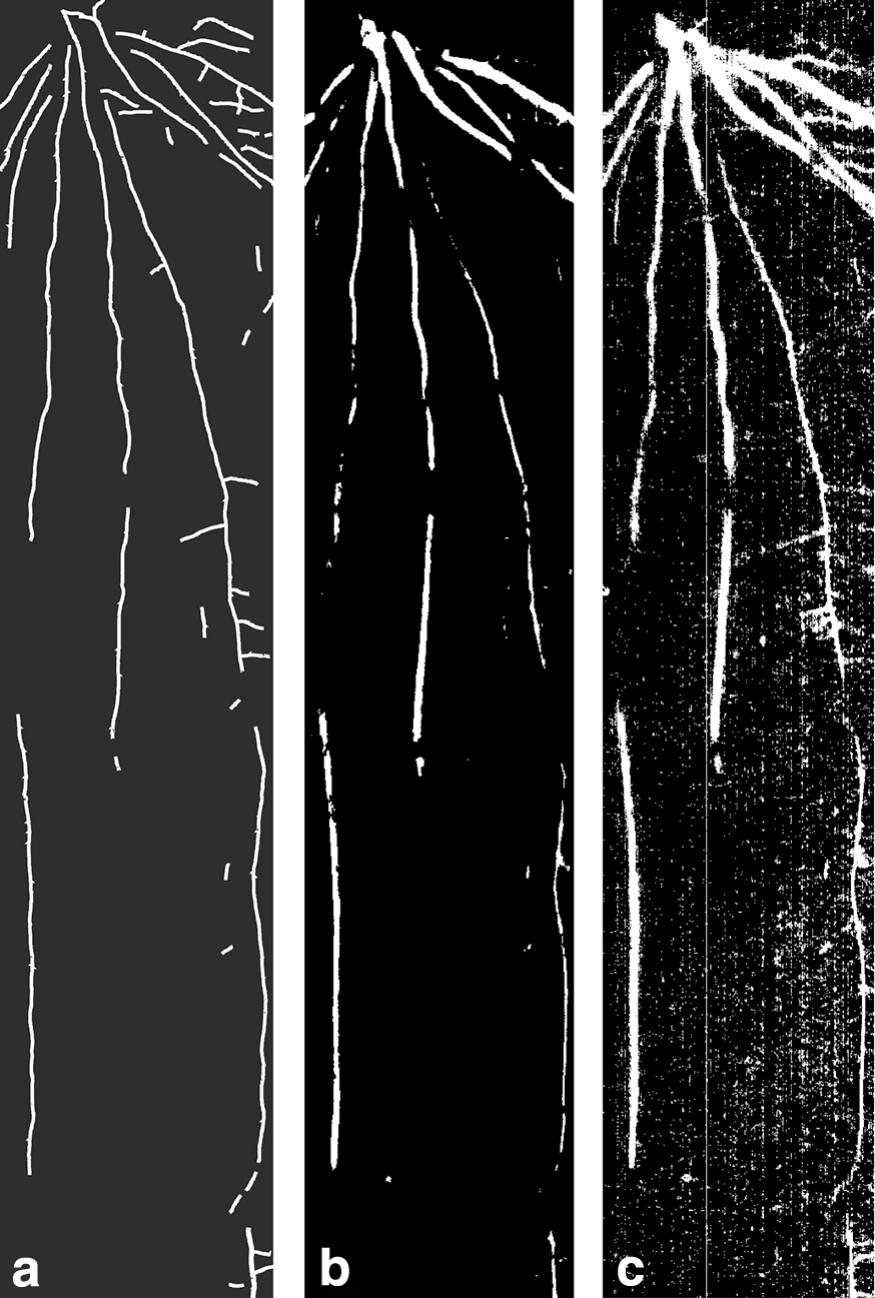
Root image at 1450 nm with manually tracked roots (**a**), segmented via fuzzy clustering (**b**) and multilevel thresholding (**c**)

In the topsoil overlap between different strategies was comparatively large, i.e. similar length estimates and thereby accuracy in relation to the manually tracked reference length could be obtained via different image processing strategies. In the brighter subsoil differences were substantially higher and only some processing strategies resulted in segmented root length near to the reference length. Overall fuzzy clustering tended to be more conservative, while thresholding resulted in high number of pixels classified as roots with a tendency to increase noise from misclassified pixels. This is exemplified in Fig. 5: compared to the manually tracked reference (Fig. 5a) it can be seen that fuzzy clustering (Fig. 5b) did not capture some lateral root axes, while thresholding (Fig. 5c) resulted in a noisier image still conserving these lateral axes.

The best segmentation results with root length next to the manually tracked reference and low image noise were obtained for the topsoil with log-linearization and ALS correction as pre-treatment and using thresholding on the single most informative band (difference to manual tracking 3.5%). For the subsoil log-linearization and ALS correction in combination with fuzzy clustering on the single most informative band resulted in the lowest difference to manual tracking (4.6%).

### Spectral versus colour based root segmentation

**Fig. 6:**
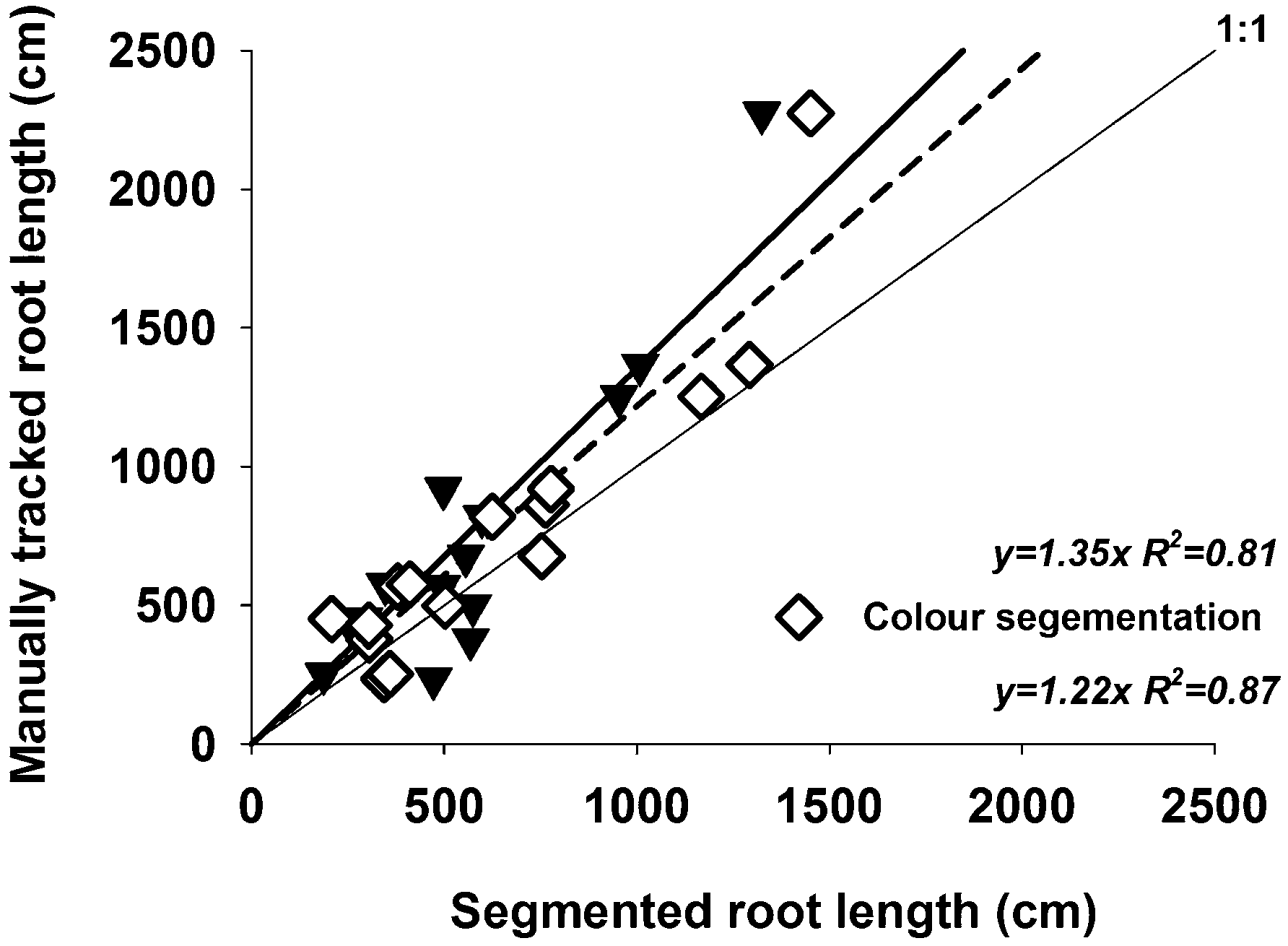
Prediction of manually traced visible root length from automatic spectral and colour segmentation methods

The topsoil and subsoil processing strategies with best segmentation result were then applied to the entire rhizobox images and compared to colour threshold segmented RGB images as well as manually tracked references. Figure 6 shows that both, spectral and colour based image segmentation, reliably predicted visible root length. The spectral approach captured slightly less root length (77.0%) compared to colour based segmentation (83.4%). However, slopes of both segmentation methods were not significantly different (*P* = 0.225) indicating that both methods had similar performance to predict visible root length for substrates used here.

Overall root length was significantly influenced by the soil material (807.3 cm for topsoil vs. 461.7 cm for subsoil; *P* = 0.012) and soil moisture (884.2 cm for moist soil vs. 423.1 cm for dry soil; *P* < 0.001), but not by segmentation method (770.6 cm for manual tracking, 593.5 cm for automated spectral segmentation, 643.0 for automated RGB segmentation; *P* = 0.377) nor interaction between segmentation method and treatment.

On average visible root axes at the observation window, accessible to optical imaging, were 27.3% of total root length in the rhizoboxes. The relation between visible and total root length was significant with an *r*^*2*^ of 0.74 (*P* < 0.001; *cf.* Additional file 5). For the topsoil, surface visibility was significantly higher in the control compared to the dry treatment (37.6% vs. 15.6% of all root length visible at the glass observation window; *P* = 0.030), while it did not differ in the subsoil.

### Spatial differentiation of root spectral pattern

A key advantage of hyperspectral imaging is the chemometric information contained in the spectral images. Radial differentiation in spectral signatures from center to border (*cf.* Additional file 6) were predicted by a decision tree model with an *r* = 0.86 for the training data and *r* = 0.48 for the validation data. Table 4 gives the percentage of pixels allocated to the respective labelled classes by the trained model.

**Table 4:** Predicted allocation of root pixels to labelled radial classes from root centre (inner 0–0.1 mm) to root border (> 1.3 mm) based on their spectral characteristics using a decision tree model

Class labels (mm)	Predicted allocation to class (%)							
	0–0.1	0.1–0.3	0.3–0.5	0.5–0.7	0.7–0.9	0.9–1.1	1.1–1.3	> 1.3
0–0.1	51.1	12.0	7.4	7.5	8.4	8.9	9.5	8.8
0.1–0.3	32.6	68.2	27.4	27.4	29.0	29.7	34.3	31.7
0.3–0.5	13.2	15.8	58.4	26.2	28.0	29.7	26.6	31.1
0.5–0.7	2.8	3.6	6.2	38.1	8.3	8.2	8.0	7.6
0.7–0.9	0.2	0.3	0.6	0.7	26.2	1.0	1.1	0.9
0.9–1.1	0.0	0.0	0.1	0.1	0.0	22.5	0.1	0.1
1.1–1.3	0.0	0.0	0.0	0.0	0.0	0.0	20.4	0.0
> 1.3	0.0	0.0	0.0	0.0	0.0	0.0	0.0	19.8
Percentage total pixels	18.9	39.2	26.1	10.4	3.3	1.2	0.5	0.3

Percentage of root pixels in the respective class in relation to the total number of root pixels is given in the bottom line

**Fig. 7:**
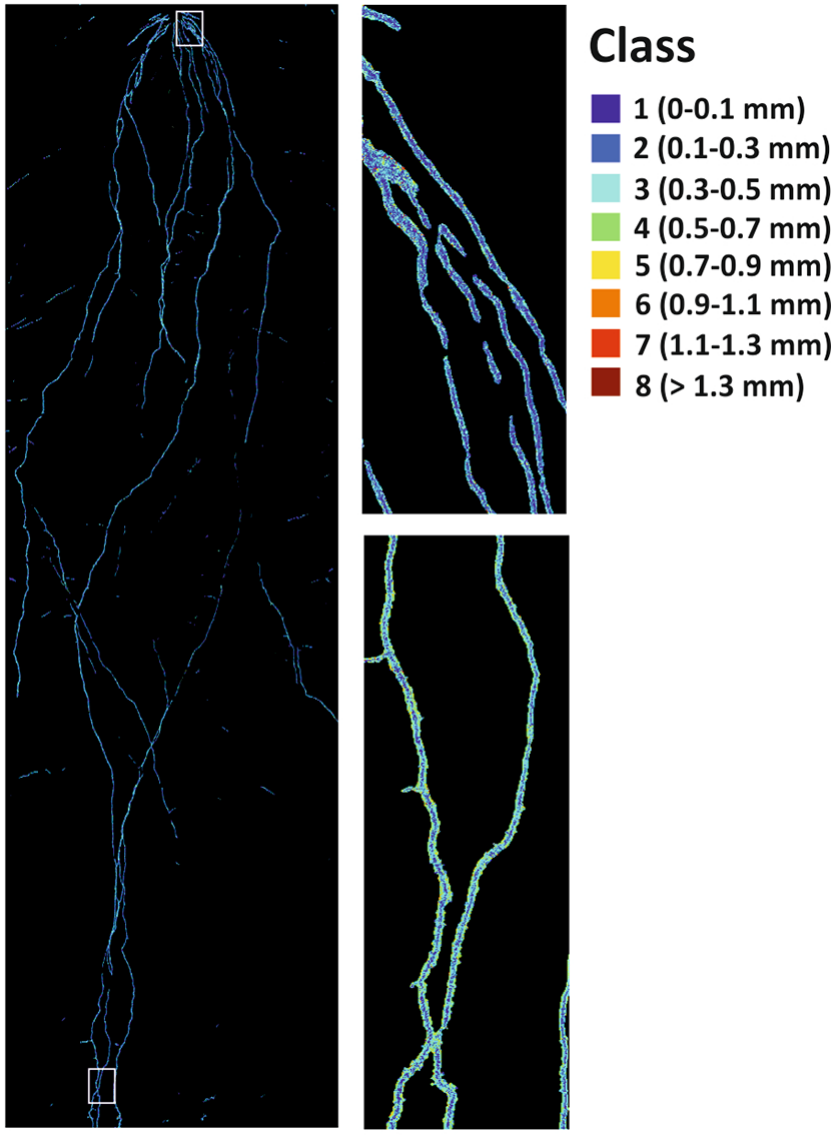
Radial pattern of root spectral reflectance classified with a decision tree model trained for eight radial classes from center to border at 0.2 mm increment. The white frames on the whole root image indicate the location of top and bottom close-up sections

The chemometric model identified two main spectrally distinctive regions: an inner region (0–0.3 mm) where in average 82% of the pixels are allocated to the first and second class, and an outer region where in average 62% of the pixels are allocated to spectral classes > 0.3 mm. Within these two regions, the spectral pattern of pixels largely overlapped. It should be noticed that coarser root axes (> 0.7 mm) made up only 5.3% of all root pixels in the image. Figure 7 shows the classification result with close-ups for the basal and apical region. At the basal parts of root axes pixels with the spectral features of the central classes (dark blue) are more abundant compared to the apical parts with predominance of pixels with spectral characteristics of the outer region (light blue).

### Temporal changes of spectral signature of decaying roots

**Fig. 8:**
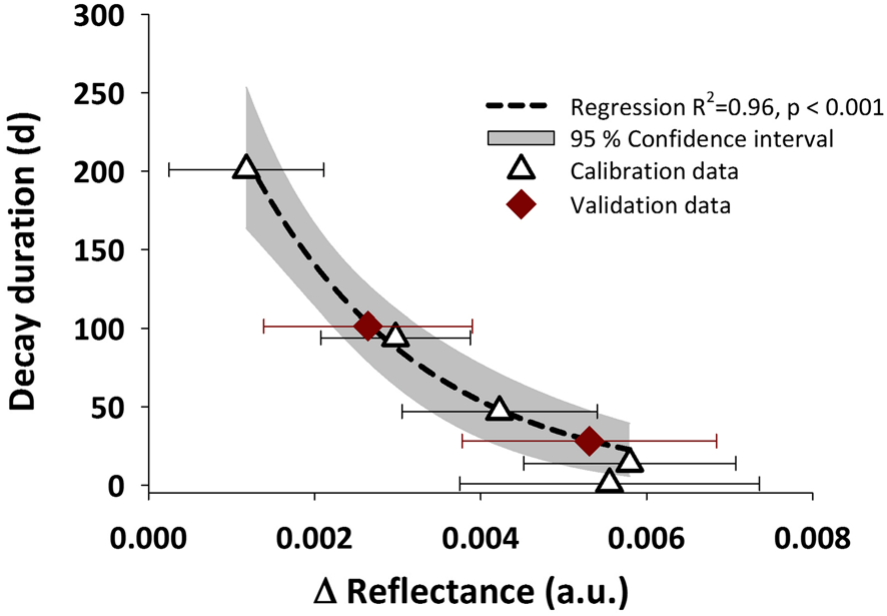
Relation between spectral reflectance (first derivative difference spectra 1649–1447 nm) and decay duration (days after clipping of the shoot)

Differentiation in the spectral pattern between the initial and final time was highest for the first derivative difference spectra between wavelengths of 1649–1447 nm with a maximum Bhattacharyya distance of 2.21 (Additional files 7 and 8). Figure 8 shows the exponential model predicting root decay duration as a function of spectral reflectance with two validation time points (28 and 101 days after clipping) not included in curve fitting. Changes in spectral reflectance were closely related to decay duration (*r*^*2*^ = 0.96).

**Fig. 9:**
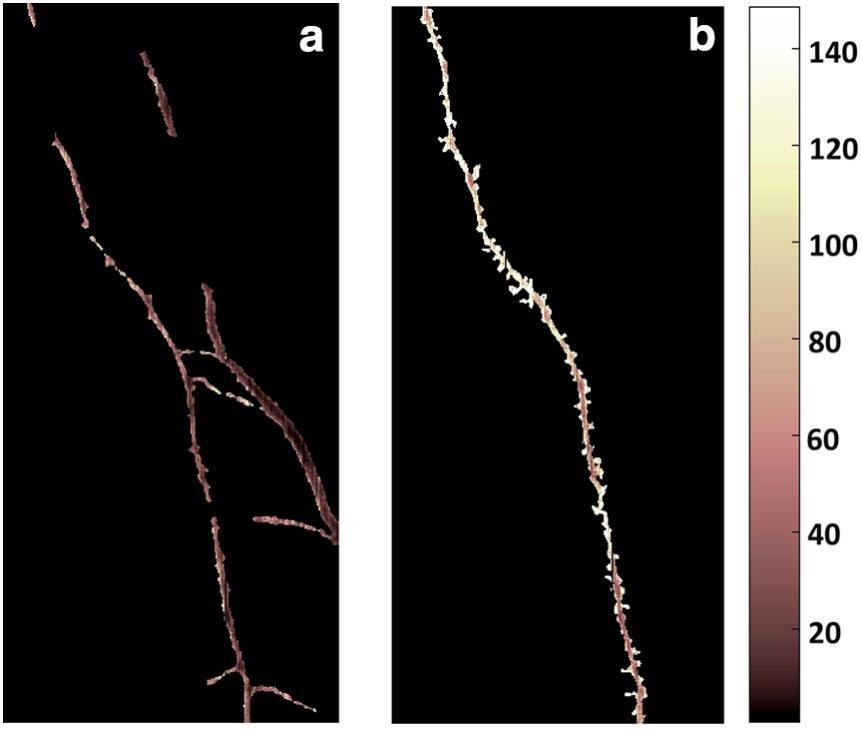
Close-up of the same image region with fine-mapping of regression model for root decay duration (*cf.* Fig. 8) on root pixels; **a** root image at 28 days after clipping, **b** root image at 101 days after clipping. Colour scale: days after clipping

Figure 9 provides a close-up image of root axes at 28 and 101 days after clipping with mapping of the decay model on the root pixels. At 101 of root decay (Fig. 9b) only one major root axes was recognized (segmented) as spectrally different from the soil background, while the coarser shoot-borne root axis and the laterals were apparently strongly decomposed. On the contrary a small part of the main axis was not recognized in the image at 28 days after clipping (Fig. 9a). Fine mapping also revealed a centre-to-border gradient of reflectance values, suggesting chemometric similarity between spatial (radial) and temporal spectral patterns.

## Discussion

### Rhizobox experimental system

Inference from root phenotyping towards field environments critically depends on suitable substrates [24] and plant development stage, particularly for monocots with shoot-borne roots emerging upon tillering [74, 80]. The rhizobox setup used in this study represents a field-near system with soil as substrate and sufficiently large containers for unconstraint (cereal) root growth until end of vegetative development. Size of phenotyping systems is particularly important when focussing on root contribution to drought resistance [52, 56]. The rhizoboxes of 1 m height used here closely match the hydraulic behaviour of similarly textured field soils (e.g. Saxon and Rawls [60]: field capacity for silt loam 0.31 cm^3^ cm^−3^; *cf.* Table 3: rhizobox drained upper limit 0.27–0.33 cm^3^ cm^−3^).

### Imaging setup

Camera model and spectrograph determine the spectral range as well as spectral and spatial resolution of imaging systems. For root imaging high spatial resolution is required as most root axes of annual plants are allocated in the lowest diameter classes (very fine < 0.5 mm; fine 0.5–2 mm) according to Böhm classification [7]; e.g. here 48.7% < 0.5 mm and 99.8% < 2 mm. The spatial resolution limit of the scanning device used here is 0.1 mm. This is only slightly lower compared to common settings of RGB root scanning (400 dpi equivalent 0.063 mm pixel size; Himmelbauer et al. [25]) and μCT (0.056–0.099 mm according to pot size; Metzner et al. [42]). Image acquisition time for a rhizobox of 100 cm height an 30 cm width at this spatial resolution is 16 min. This is substantially shorter to high-resolution 3D methods (e.g. 20 min for a 30 cm high × 8.1 cm inner diameter tube; Metzner et al. [42]) and therefore suitable to mature root phenotyping.

Compared to RGB imaging, a hyperspectral approach substantially increase data size as well as the complexity of evaluation. The automated segmentation pipeline presented here however allows a comparatively rapid data processing to obtain a binary root image (about 40 min). Still comparison of RGB versus spectral segmentation suggested an advantage of NIR bands just in case of very bright soil background. Otherwise VIS bands are suitable for detection of living root axes from soil background [49] with high resolution RGB cameras leading to a potentially superior root-soil segmentation compared to a NIR setup (see discussion on spectral vs. colour based segmentation results below).

Thus the most relevant added value of hyperspectral NIR imaging is related to the physicochemical information contained in this wavelength range. These chemical imaging capacities of a hyperspectral setup are determined by spectral range and resolution. The camera sensor of the device used here allows imaging between 900 to 1700 nm with 256 narrow bands (i.e. spectral resolution of 3.1 nm) covering wavelength bands such as water, cellulose, lignin, starch and protein [15, 30, 64, 70]. Hyperspectral imaging thus has the potential to extend root phenotyping towards such physicochemical root zone properties with relevance for root functionality.

### Image processing strategies for feature detection

Exploitation of the information hidden in hyperspectral data critically depends on algorithms capable to detect the features of interest. Root phenotyping, similarly to food quality control, targets biological objects of variable biochemical composition and high tissue water content. Additional challenges in root–soil image processing arises from the complex background with uneven surface morphology from soil aggregates, and non-uniform and time-variable water content. Esquerre et al. [16] suggested chemometric image pre-treatment to suppress surface morphology and improve contrast between image features. Also our results demonstrated that root versus soil pixels could be better separated after spectral pre-treatment. Baseline correction with asymmetric least squares and the use of difference spectra were particularly efficient for enhancing feature contrasts. A major disadvantage of ALS baseline correction is the high computational time required for large datasets (1545.5 s for a 9.4 GB image) compared to other pre-treatment methods with similar performance such as first derivatives (33.4 s) or standard normal variate (6.8 s) of log-linearized spectra.

Beyond visual inspection, different selection methods can be applied for an objective pre-treatment selection and extraction of the most informative wavelengths. Although the different measures (non-Gaussian indicators, Bhattacharyya distance) did not point to a unique wavelength to maximise feature contrast, for both topsoil and subsoil foreground (root)–background (soil) distinction was highest between 1400 and 1480 nm. Secondary regions of distinctive wavebands occurred between 1050–1090 nm and 1620–1700 nm. The region around 1450 nm corresponds to a major water absorption band (e.g. [20]). For leaf tissues Mobasheri and Fatemi [44] also found high correlation to equivalent water thickness around 1050 nm. In the third region of high root-soil contrast several spectral peaks for cellulose (1632 nm), hemicellulose (1668, 1681 nm) and lignin (1672–1674, 1677, 1685 nm) are located [62].

Segmentation accuracy was dependent on soil substrate and pre-treatment. The most efficient pre-treatment for both substrates was log-linearization and ALS correction, while thresholding was most accurate in the topsoil and fuzzy clustering in the subsoil.

Unsupervised approaches such as clustering can be readily applied in automated segmentation of phenotyping data as they do not require a user-labelled training dataset. Still clustering results depend on the user-defined cluster number which implies a certain degree of subjectivity and empiricism [28]: e.g. using two clusters only to represent the data structure, one for root and one for soil, tended to increase noise in segmentation results, while higher cluster number underestimated root length by allocating root pixels to a mixed class. Stability measures can provide some decision-support to assess the clustering result [32]. However, also clustering algorithm and distance metrics influence the segmentation result. Thus optimizing the settings of unsupervised approaches before evaluation of an image series still requires some user interaction.

Supervised machine learning approaches are often considered as particularly adapted for classification of multidimensional data [66, 79]. However, our results did not suggest an advantage of using support vector machine segmentation. Belgiu and Drǎguţ [3] and Anzanello et al. [2] concluded that transformation of spectral data could change the superiority of supervised versus unsupervised approaches. Other studies (e.g. [11]) reported a slightly better performance of supervised classification. Chen and Stow [9] underlined that size and homogeneity of training data are critical for the accuracy of supervised approaches. In our case the labelled data were only 0.6% of image pixels. Also inaccuracy of freehand-labelling could have reduced performance: considering the tiny size of root axes and constraints of camera resolution falsely labelling of soil pixels as root and vice versa, or influence of mixed border pixels can hardly be excluded [55]. Therefore unsupervised approaches were more efficient for automatic segmentation in a phenotyping context, while supervised machine learning approaches were most adapted to subsequent chemometric analyses.

Overall our results demonstrate that an adequate image processing strategy has to be found for an experimental dataset with a specific foreground (species dependent roots)–background (soil) combination. There is no unique combination of pre-treatment, band selection and segmentation algorithms that fits all hyperspectral root images. However, following the image processing pipeline presented here, the appropriate pre-treatment and segmentation methods and best bands for an optimum segmentation result can be found efficiently.

### Spectral versus colour based segmentation

Our results demonstrated that even for bright subsoil segmentation via colour thresholds did not differ in performance compared to spectral segmentation. Overall the colour based segmentation was even slightly, though not significantly, more accurate than the spectral segmentation. We hypothesize that the main reason for this was the smaller pixel size of high resolution RGB cameras (6.6 μm for the camera model used here) better capturing fine lateral axes. Still there could be substrate types such silica sand (Additional file 9) where colour based segmentation fails, while still spectral patterns allow foreground–background distinction. Also more advanced segmentation algorithms (e.g. sub-pixel mapping approaches; e.g. [41, 71, 77] might further improve detection of tiny structures with the given spatial resolution of the camera.

Importantly all reports on rhizobox-like systems found a significant relation between visible and total root length [47]. Also here total root length could be predicted from visible axes (*r*^*2*^ = 0.74) with a similar percentage of visible roots as reported by Pfeifer et al. [53] for barley (between 20 and 30%) in a comparable setup.

### Chemometric root analysis

Chemometric classification of the segmented root axes with a decision tree model revealed major spectral distinction between a central region extending to an average radius of 0.3 mm and the region beyond 0.3 mm to the outer border of root axes. It is hypothesized that the model captured distinctive constituents of the root stele via their spectral pattern (particularly related to water absorption; *cf.* Additional file 6). For example Watt et al. [73] reported stele diameter of cereal primary roots in the range of 0.1–0.2 mm.

Spectral root signatures changed with root decay time after clipping. The temporal change was most pronounced at a wavelength difference of 1649 minus 1447 nm. The region around 1649 nm contains several bands for cellulose, hemicellulose and lignin [35, 62], while the region around 1447 nm has strong water absorption properties [20]. It is thus suggested that the spectral pattern could indicate a change in tissue water content relative to the concentration in structural carbohydrates. The dynamics of changing spectral reflectance were modelled by an exponential curve which is common in plant (root) litter decomposition [14, 65]. Fine-mapping of the model on root pixels revealed that spatial (mainly radial) differences of root constituents were encoded by the same spectral signature as decay duration. Upon calibration with measured root chemical components (e.g. C:N, lignin, cellulose, hemicellulose, calcium; [78]) it can be expected that hyperspectral imaging provides relevant insights into the spatial and temporal biochemical patterns of plant roots and a better understanding of root functioning.

## Conclusions

Hyperspectral imaging is a novel approach for root phenotyping of soil grown plants. Although acquisition and processing time limits throughput compared to RGB imaging, spectral signatures provide potential added-value by encoding physico-chemical root/soil constituents. Hyperspectral imaging thus bridges between high-throughput RGB and CT/MRI deep root phenotyping technologies. Phenotyping requires a high degree of automation in image processing. Adequate methods for root segmentation and chemometric analysis are therefore critical. With the hyperspectral image processing pipeline presented here an adapted strategy for specific experimental settings can be found efficiently. Pre-treated images were successfully segmented with unsupervised clustering and thresholding approaches. Due to lower resolution of spectral images, still there was no segmentation advantage over colour thresholding from RGB images. However using chemometric models, spectral signatures allowed to infer on distinctive radial composition of root axes and their decomposition dynamics. These first results demonstrate that the developed data processing pipeline facilitates application of hyperspectral imaging as promising technology for advances in root research. Further investigations will highlight the relevance of distinctive spectral root properties in relation to root functionality. Finally, exploration of large-size hyperspectral root data will contribute to define target wavelengths relevant for the design of multispectral, high-throughput structural–functional root phenotyping systems.

## Additional files

**Additional file 1.** Soil water retention curves of top- and subsoil.

**Additional file 2.** Example of different pre-treatment evaluation and band selection approaches.

**Additional file 3.** Movie over all wavelength for topsoil image with log-linearization and ALS baseline correction.

**Additional file 4.** Relation between segmented root length and image noise.

**Additional file 5.** Relation between visible and total root length.

**Additional file 6.** Raw spectra of root pixels from center to border region.

**Additional file 7.** Untransformed and first derivative spectra during root decay.

**Additional file 8.** Histograms at wavelengths with maximum separation between initial and final decay time for different pre-treatments.

**Additional file 9.** RGB image of sand grown root system with spectral segmentation and manual tracking.
