# Supplementary material for: Hyperspectral imaging: a novel approach for plant root phenotyping
Source: Plant Methods. 2018 Oct 3;14:84. doi: 10.1186/s13007-018-0352-1 (PMC6169016; doi:10.1186/s13007-018-0352-1)
Supplement: Supplementary file 1 — Additional file 1. Soil water retention curves of top- and subsoil. [file 13007_2018_352_MOESM1_ESM.docx]

**Additional File 1:** Soil water retention curves of top- and subsoil.

**
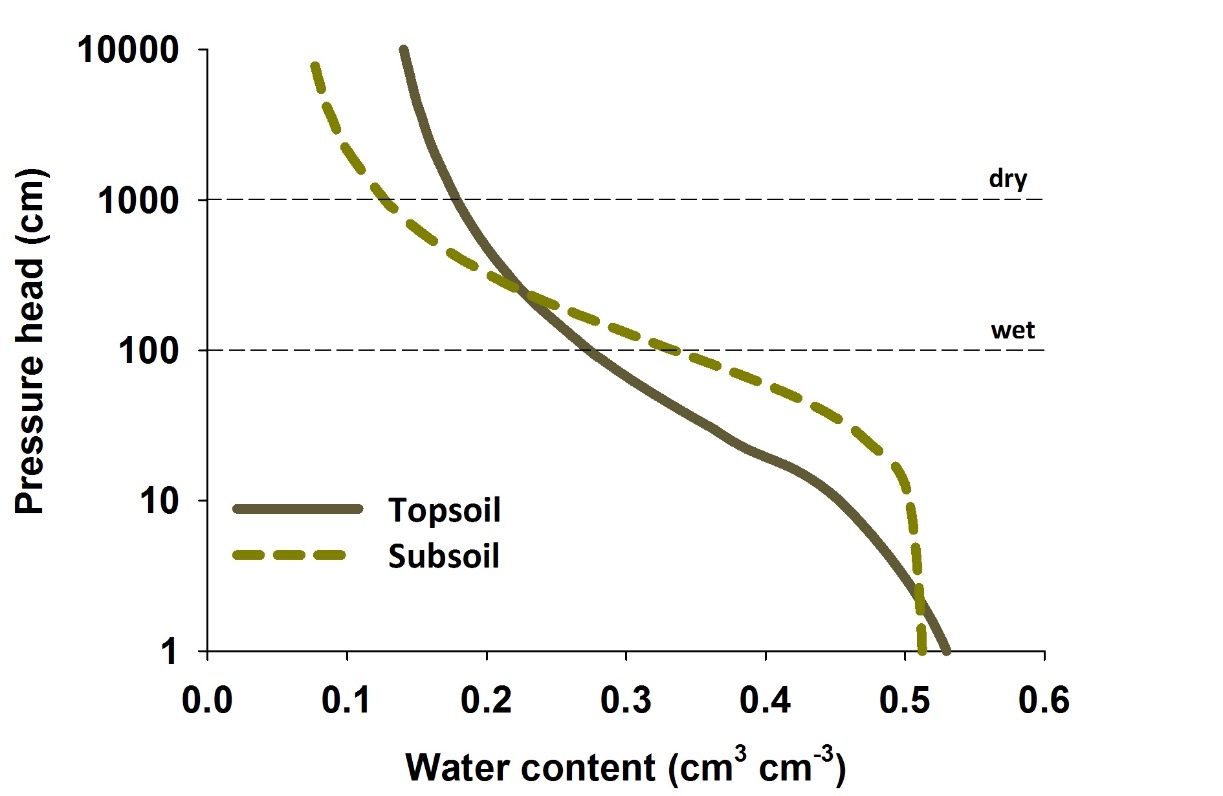
**

**Additional File 1:** Soil water retention curves of topsoil and subsoil measured by evaporation method (Hyprop, METER Group, Inc. USA ). Wet and dry indicate the water content for the two moisture treatments, where wet is equal to field capacity (drained upper limit for a rhizobox of 100 cm height).
