# Supplementary material for: Hyperspectral imaging: a novel approach for plant root phenotyping
Source: Plant Methods. 2018 Oct 3;14:84. doi: 10.1186/s13007-018-0352-1 (PMC6169016; doi:10.1186/s13007-018-0352-1)
Supplement: Supplementary file 2 — Additional file 2. Example of different pre-treatment evaluation and band selection approaches. [file 13007_2018_352_MOESM2_ESM.docx]

**Additional File 2:** Example of different pre-treatment evaluation and band selection approaches.

**
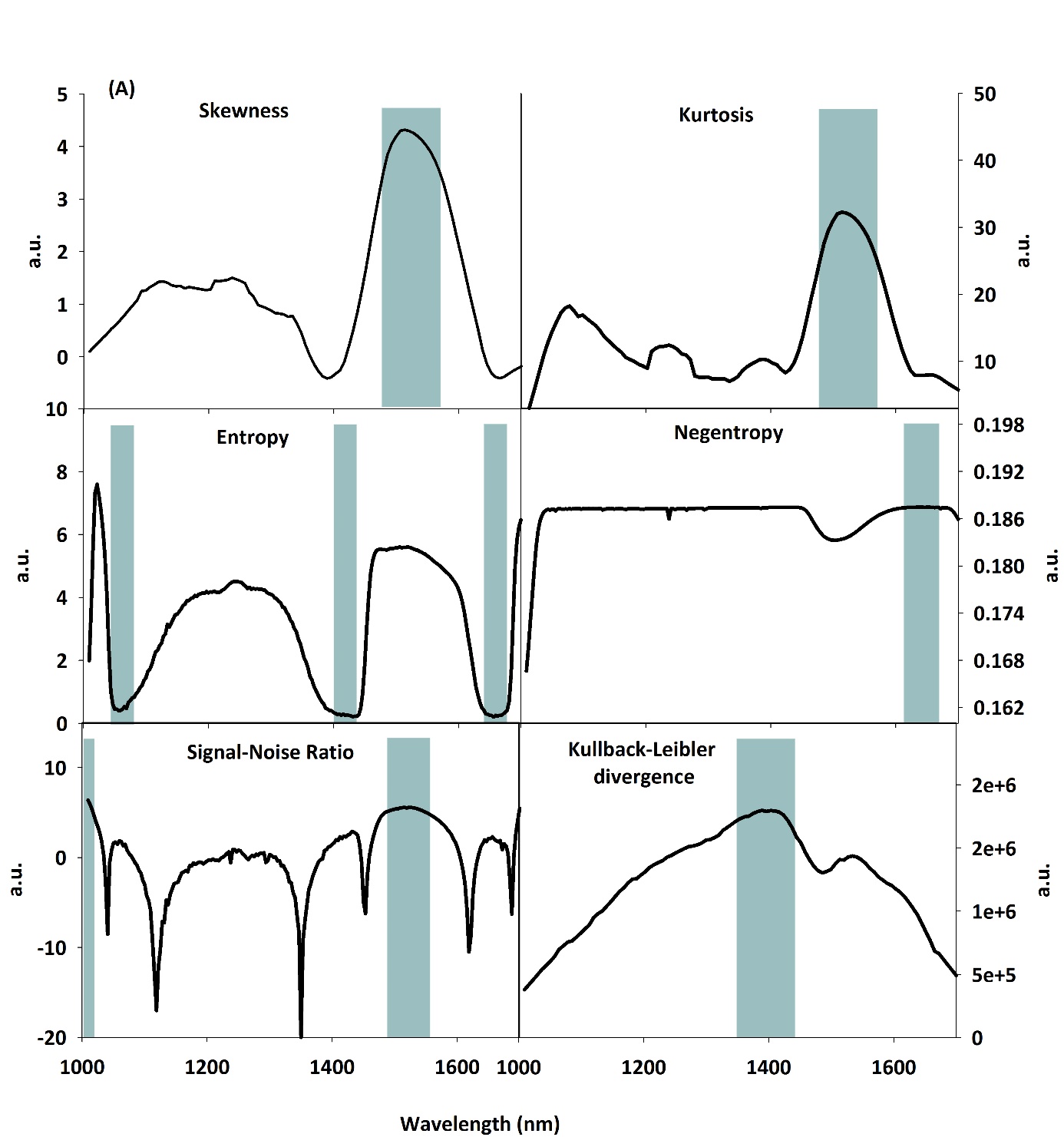
**

**Additional File 2a** Example of Non Gaussian measures indicating wavebands with high information content on the whole image (blue area show the bands in the upper 5 % quantile). a.u. arbitrary unit.

**
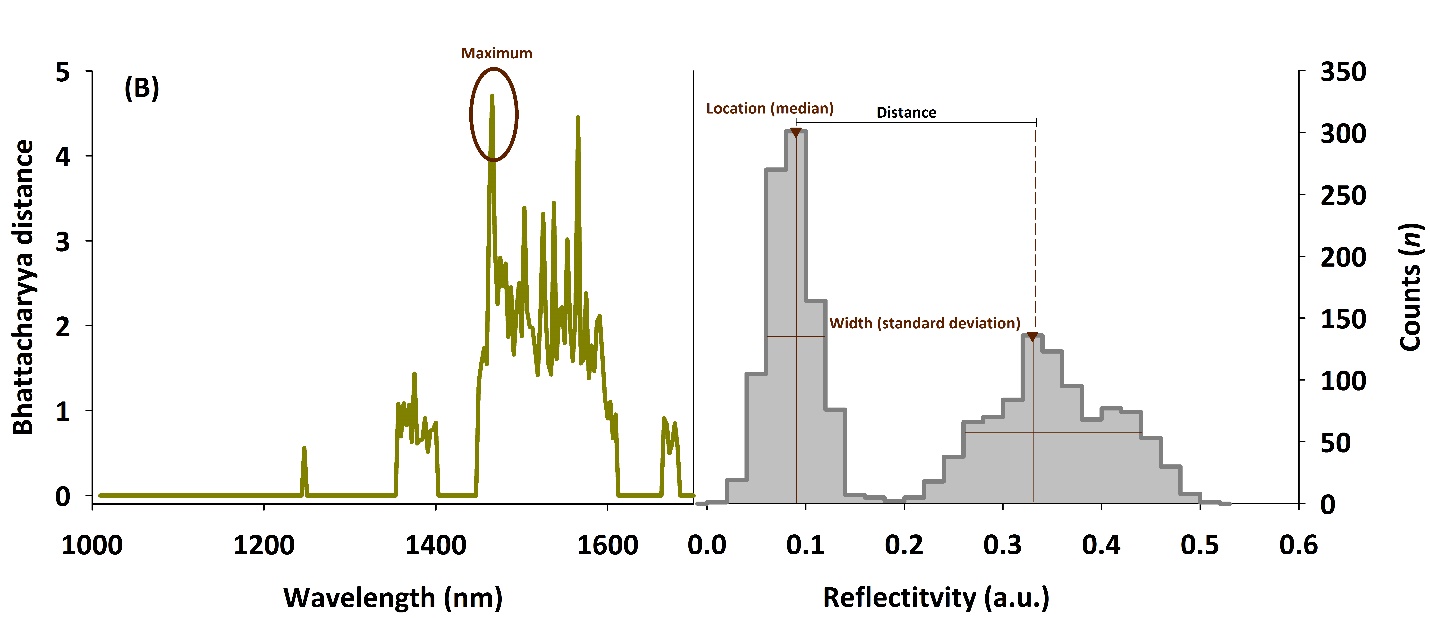
**

**Additional File 2b** Example of Bhattacharyya distance for single spectra (left) as an indicator of the spectral distinctness between root and soil pixel obtained via histogram evaluation (right).

**
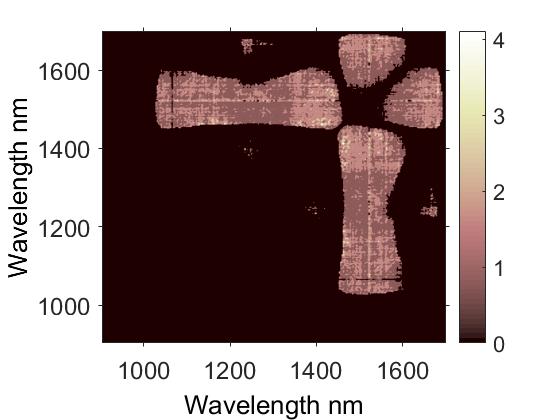
**

**Additional File 2c** Identification of wavelengths with maximum Bhattacharyya distance for difference spectra (λ_m_- λ_n_ with m,n= 1…222).
