# Supplementary material for: Hyperspectral imaging: a novel approach for plant root phenotyping
Source: Plant Methods. 2018 Oct 3;14:84. doi: 10.1186/s13007-018-0352-1 (PMC6169016; doi:10.1186/s13007-018-0352-1)
Supplement: Supplementary file 4 — Additional file 4. Relation between segmented root length and image noise. [file 13007_2018_352_MOESM4_ESM.docx]

**Additional File 4** Relation between segmented root length and image noise.


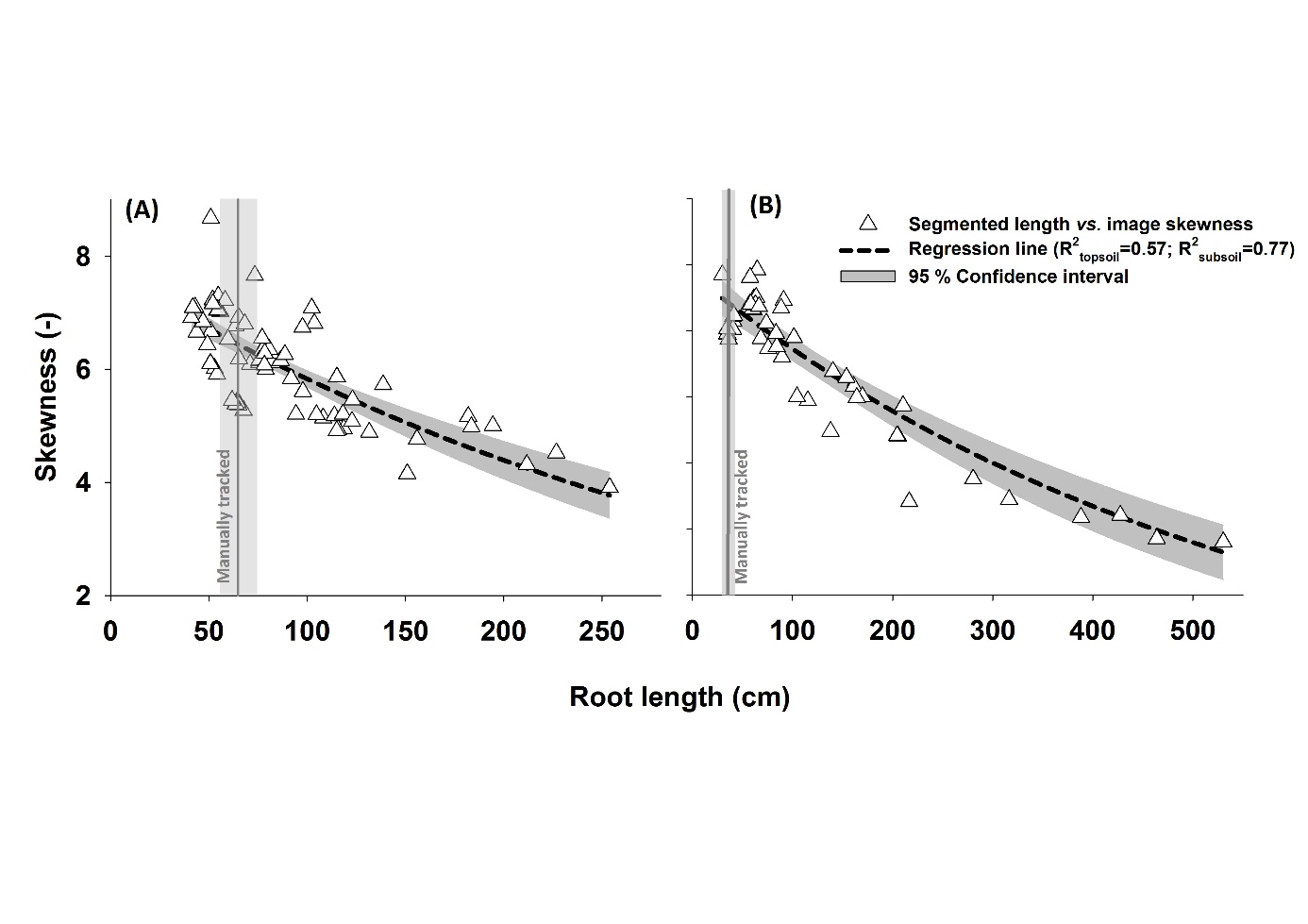


**Additional File 4** Relation between segmented root length and image noise expressed via skewness for topsoil (A) and subsoil (B).
