# Supplementary material for: Hyperspectral imaging: a novel approach for plant root phenotyping
Source: Plant Methods. 2018 Oct 3;14:84. doi: 10.1186/s13007-018-0352-1 (PMC6169016; doi:10.1186/s13007-018-0352-1)
Supplement: Supplementary file 5 — Additional file 5. Relation between visible and total root length. [file 13007_2018_352_MOESM5_ESM.docx]

**Additional File 5** Relation between visible and total root length.**
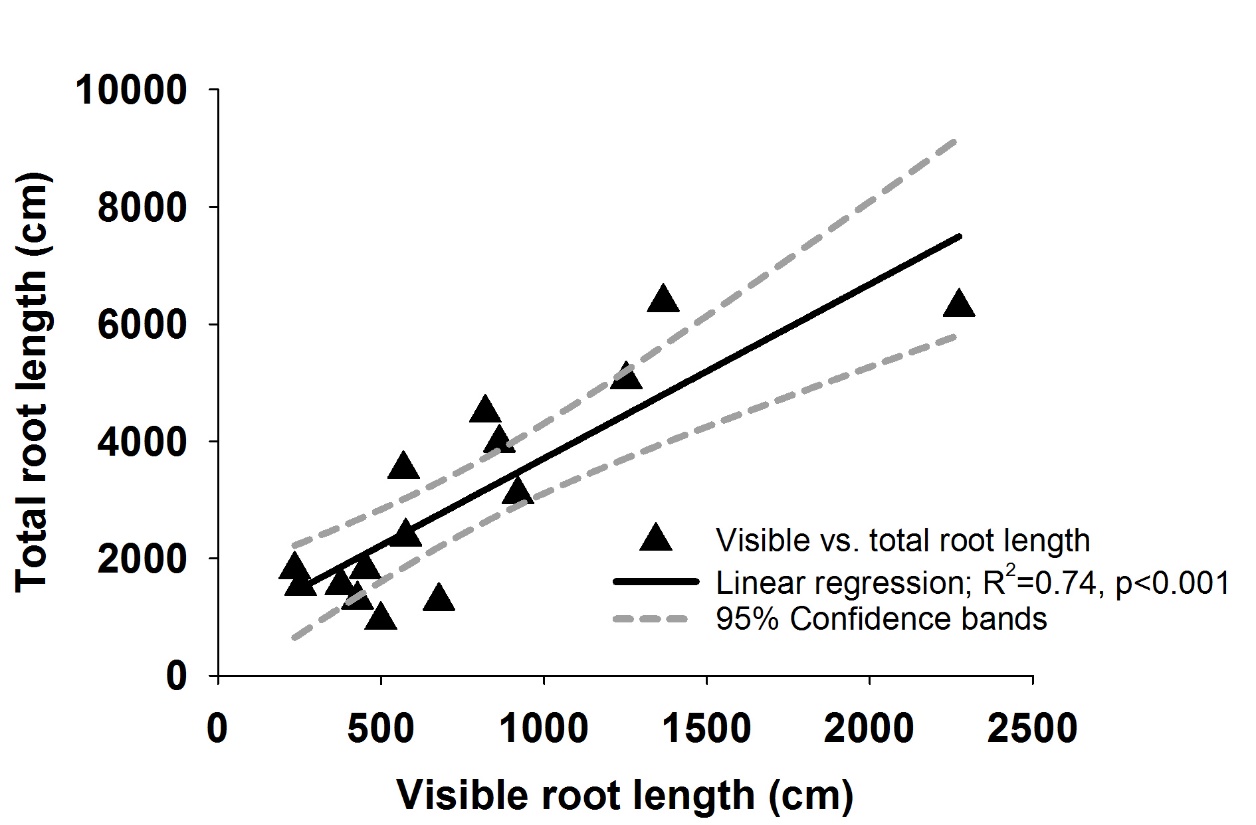
**

**Additional File 5** Relation between visible root length on the observation window (accessible to imaging) and total root length obtained after opening the rhizoboxes and separating roots from soil.
