# Supplementary material for: Hyperspectral imaging: a novel approach for plant root phenotyping
Source: Plant Methods. 2018 Oct 3;14:84. doi: 10.1186/s13007-018-0352-1 (PMC6169016; doi:10.1186/s13007-018-0352-1)
Supplement: Supplementary file 6 — Additional file 6. Raw spectra of root pixels from center to border region. [file 13007_2018_352_MOESM6_ESM.docx]

**Additional File 6** Raw spectra of root pixels from centre to border region.

**
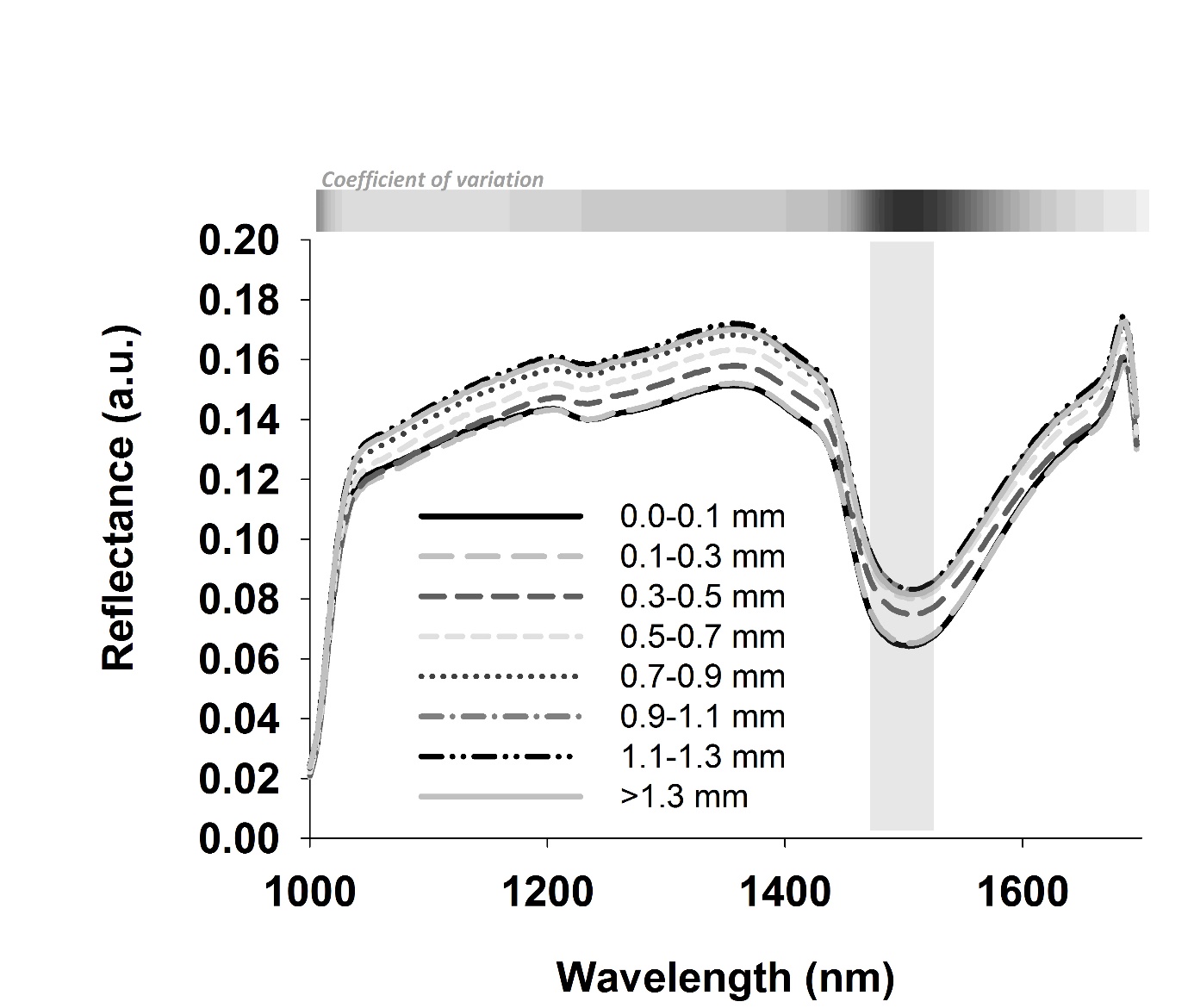
**

**Additional File 6** Raw spectra of root pixels from centre (0-0.1 mm) to border region at 0.2 mm increment. Differentiation is indicated by the coefficient of variation (CV) over the measured wavelength region (horizontal bar; light grey low CV, black high CV). The vertical grey bar indicates the region with highest CV.
