# Supplementary material for: Hyperspectral imaging: a novel approach for plant root phenotyping
Source: Plant Methods. 2018 Oct 3;14:84. doi: 10.1186/s13007-018-0352-1 (PMC6169016; doi:10.1186/s13007-018-0352-1)
Supplement: Supplementary file 7 — Additional file 7. Untransformed and first derivative spectra during root decay. [file 13007_2018_352_MOESM7_ESM.docx]

**Additional File 7** Untransformed and first derivative spectra during root decay.

**
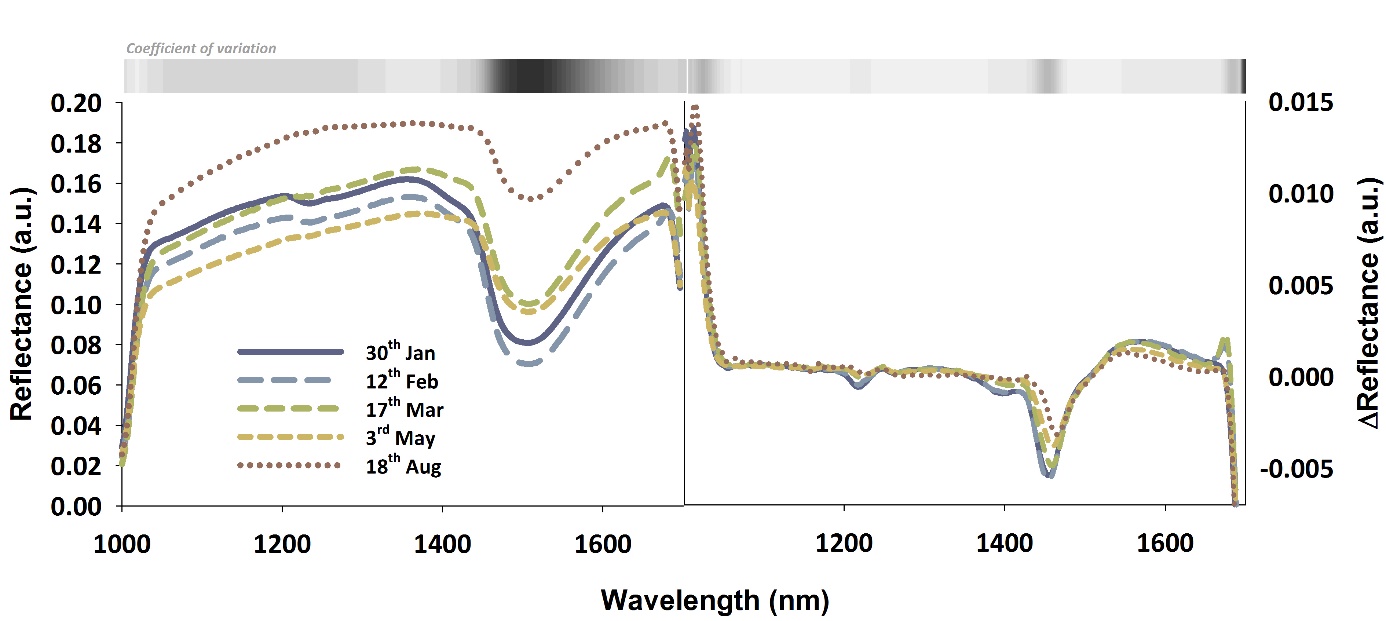
**

**Additional File 7** Untransformed (left) and first derivative (right) mean spectra of roots at different dates after clipping of the shoot on 30th of January. The grey shaded area at the top shows the coefficient of variation indicating the wavelength region with highest distinction between the spectral lines.
