# Supplementary material for: Hyperspectral imaging: a novel approach for plant root phenotyping
Source: Plant Methods. 2018 Oct 3;14:84. doi: 10.1186/s13007-018-0352-1 (PMC6169016; doi:10.1186/s13007-018-0352-1)
Supplement: Supplementary file 8 — Additional file 8. Histograms at wavelengths with maximum separation between initial and final decay time for different pre-treatments. [file 13007_2018_352_MOESM8_ESM.docx]

**Additional File 8** Histograms at wavelengths with maximum separation between initial and final decay time for different pre-treatments.

**
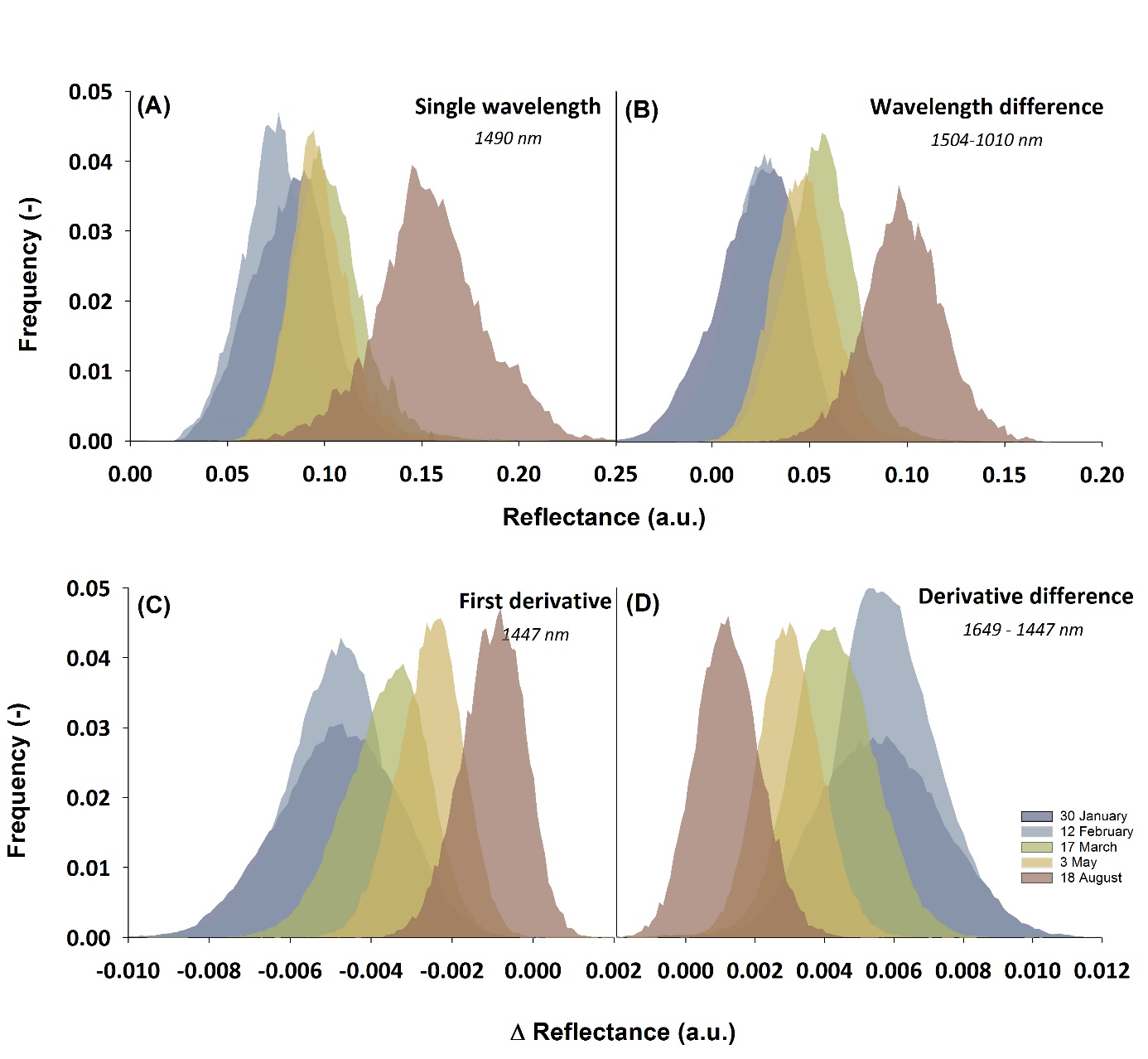
**

**Additional File 8** Histograms showing distribution of spectral reflectance values at the waveband with highest differentiation according to Bhattacharyya distance. (A) untransformed spectra, (B) difference spectra, (C) first derivative and (D) difference first derivative spectra.
