# Supplementary material for: Hyperspectral imaging: a novel approach for plant root phenotyping
Source: Plant Methods. 2018 Oct 3;14:84. doi: 10.1186/s13007-018-0352-1 (PMC6169016; doi:10.1186/s13007-018-0352-1)
Supplement: Supplementary file 9 — Additional file 9. RGB image of sand grown root system with spectral segmentation and manual tracking. [file 13007_2018_352_MOESM9_ESM.docx]

**Additional File 9** RGB image of sand grown root system with spectral segmentation and manual tracking.

**
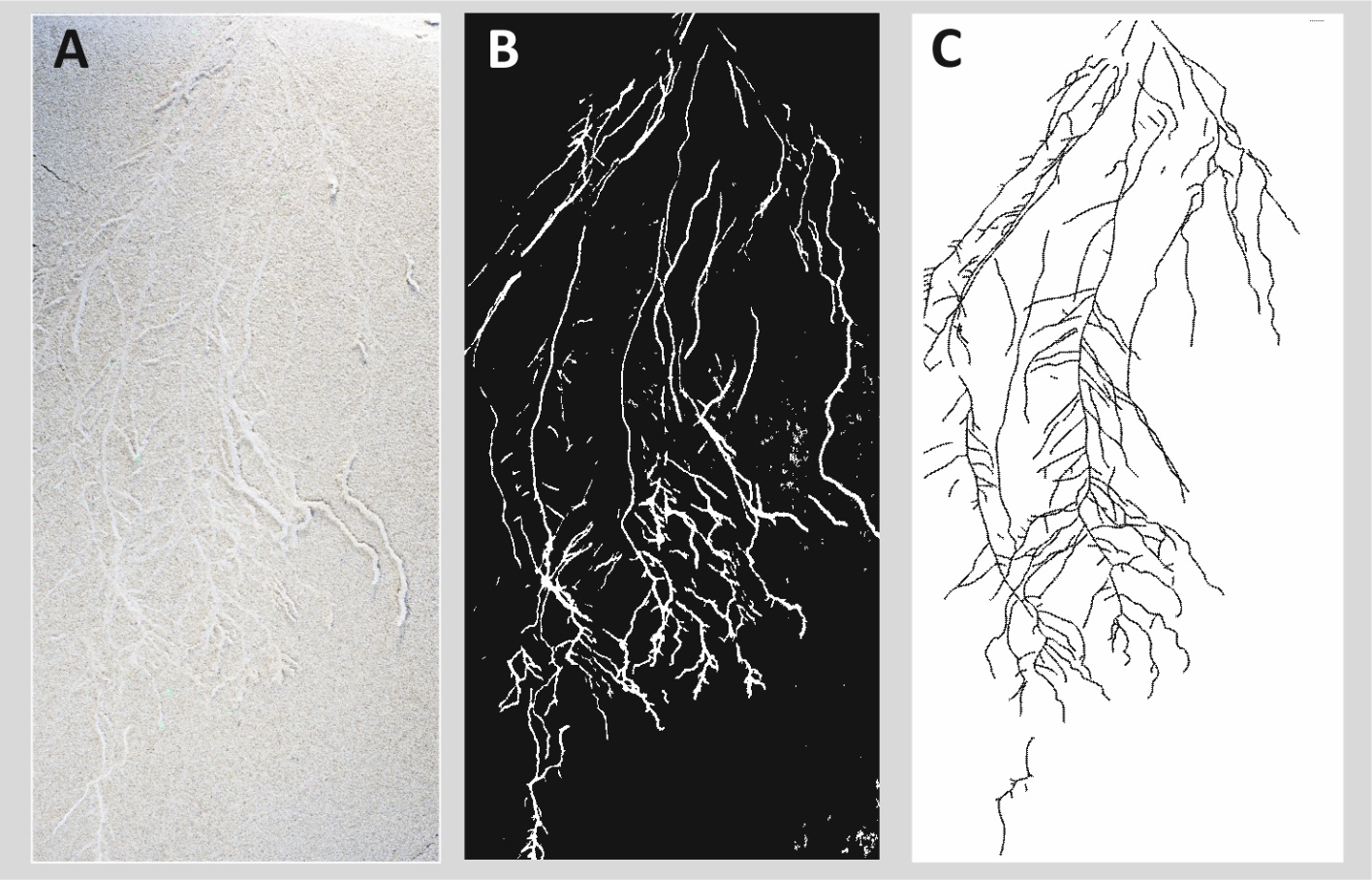
**

**Additional File 9** Root grown in washed silica sand (0.1-0.3 mm particle diameter). The RGB image (A) reveals the low contrast between roots and soil background which makes colour based segmentation impossible. Spectral segmentation however is still feasible (B; obtained via fuzzy clustering). C shows the manually tracked reference root system.
